# Prevalent bee venom genes evolved before the aculeate stinger and eusociality

**DOI:** 10.1186/s12915-023-01656-5

**Published:** 2023-10-23

**Authors:** Ivan Koludarov, Mariana Velasque, Tobias Senoner, Thomas Timm, Carola Greve, Alexander Ben Hamadou, Deepak Kumar Gupta, Günter Lochnit, Michael Heinzinger, Andreas Vilcinskas, Rosalyn Gloag, Brock A. Harpur, Lars Podsiadlowski, Burkhard Rost, Timothy N. W. Jackson, Sebastien Dutertre, Eckart Stolle, Björn M. von Reumont

**Affiliations:** 1 Justus Liebig University of Gießen, Institute for Insect Biotechnology, Heinrich-Buff-Ring 58, 35392 Giessen, Germany; 2 Department of Informatics, Bioinformatics and Computational Biology, i12, Technical University of Munich, Boltzmannstr. 3, Garching, 85748 Munich, Germany; 3 Genomics & Regulatory Systems Unit, Okinawa Institute of Science & Technology, Tancha, Okinawa 1919 Japan; 4 Protein Analytics, Institute of Biochemistry, Justus Liebig University, Friedrichstrasse 24, 35392 Giessen, Germany; 5 LOEWE Centre for Translational Biodiversity Genomics (TBG), Senckenberganlage 25, 60325 Frankfurt, Germany; 6 Fraunhofer Institute for Molecular Biology and Applied Ecology, Department of Bioresources, Ohlebergsweg 12, 35392 Giessen, Germany; 7 Rosalyn Gloag - School of Life and Environmental Sciences, The University of Sydney, Sydney, NSW 2006 Australia; 8 Brock A. Harpur – Department of Entomology, Purdue University, 901 W. State Street, West Lafayette, IN 47907 USA; 9 Leibniz Institute for the Analysis of Biodiversity Change, Zoological Research Museum Alexander Koenig, Centre of Molecular Biodiversity Research, Adenauerallee 160, 53113 Bonn, Germany; 10 Australian Venom Research Unit, Department of Biochemistry and Pharmacology, University of Melbourne, Grattan Street, Parkville, Viktoria 3010 Australia; 11 IBMM, Université Montpellier, CNRS, ENSCM, 34095 Montpellier, France; 12 Faculty of Biological Sciences, Group of Applied Bioinformatics, Goethe University Frankfurt, Max-Von-Laue Str. 13, 60438 Frankfurt, Germany

**Keywords:** Hymenoptera venom, Bee toxins, Solitary bee venom, Proteo-transcriptomics, Genomics, Venom gene evolution, Machine learning, Melittin, Apamin, Aculeatoxins

## Abstract

**Background:**

Venoms, which have evolved numerous times in animals, are ideal models of convergent trait evolution. However, detailed genomic studies of toxin-encoding genes exist for only a few animal groups. The hyper-diverse hymenopteran insects are the most speciose venomous clade, but investigation of the origin of their venom genes has been largely neglected.

**Results:**

Utilizing a combination of genomic and proteo-transcriptomic data, we investigated the origin of 11 toxin genes in 29 published and 3 new hymenopteran genomes and compiled an up-to-date list of prevalent bee venom proteins. Observed patterns indicate that bee venom genes predominantly originate through single gene co-option with gene duplication contributing to subsequent diversification.

**Conclusions:**

Most Hymenoptera venom genes are shared by all members of the clade and only melittin and the new venom protein family anthophilin1 appear unique to the bee lineage. Most venom proteins thus predate the mega-radiation of hymenopterans and the evolution of the aculeate stinger.

**Supplementary Information:**

The online version contains supplementary material available at 10.1186/s12915-023-01656-5.

## Background

Hymenoptera (sawflies, parasitoid wasps, true wasps, ants and bees) is among the most species-rich insect groups and are of tremendous ecological and economical importance [1]. The clade also contains more venomous taxa than any other. Hymenopteran venoms are secretions composed of typically short peptides, enzymes and other proteins. All proteins within the venomous mixture are referred to as “venom proteins” whereas the term “toxin” is reserved for those associated with a direct venomous function [2, 3]. The delivery system or venom apparatus used by hymenopterans to inject venom exists in a variety of states. From its origin as an ovipositor that co-injected immunomodulatory “venom” along with eggs into plant hosts (as in extant Symphyta), it evolved into the high-pressure venom systems of majority of wasps and bees and was secondarily lost in certain bee and ant lineages [4, 5]. As a result, Hymenoptera provide an exceptional opportunity to investigate the co-evolution of toxin genes and associated anatomy within a larger clade.

Because the function of many toxin-encoding genes is relatively free from pleiotropic and epistatic complications—one gene typically encodes one toxin with a clear functional role—toxins provide an excellent opportunity for investigation of the molecular mechanisms that facilitate the evolution of adaptive traits. Advances in comparative genomics and sequencing are furthering our efforts to understand these mechanisms at the genomic level [6–10]. Nevertheless, there have been only few large comparative studies focusing on the genomic origins of toxin genes and their weaponization, mostly in snakes and few other clades such as cnidarians [9, 11–17]. The origin and evolution of hymenopteran venom genes remains rather uninvestigated.

Unsurprisingly, given their economic significance, honeybee and bumblebee venoms have received the lion’s share of toxinological attention and are among the best-characterized venoms in the animal kingdom [5, 18]. The venoms of the remaining species of the hymenopteran radiation, however, including the majority of bees, remain largely unexplored despite recent proteo-transcriptomic studies on ant and wasp species [19–22]. Where studies of lesser-known Hymenoptera have been conducted, they typically deal with single crude fractions or even individual components either due to technical limitations at the time or because of applied research focus [5, 23–25]. An exception is the recent study on aculeate venoms by Dashevsky and colleagues in which more extensively sampled venoms from aculeates were proteomically characterized and few bioactivities for each tested [26]. Nevertheless, proteo-transcriptomic studies focused on injected and functionally described components are in general rather sparse and often focus on small peptides and/or are available for only few smaller groups or single taxa of hymenopterans, such as honey bees [27], ants [28, 29], spider wasps [30] and true wasps [31]. An exception is the recent study by Robinson et al. [29], who proposed that short toxin peptides of ants, bees and wasps comprise a family of “aculeatoxins”. Their argument is predominantly based upon the similarity of manually aligned propeptide sequences; however, a phylogenetic analysis or network analysis of the sequences was not provided.

Our study represents the first taxon-wide comparative genomics analysis of aculeate venom genes, including 32 hymenopteran genomes, with a particular focus on bees. We address two key questions: (1) whether bee venoms are predominantly comprised of toxins that are novel and unique to this clade, and (2) whether single gene co-option is the major mechanism of venom gene evolution in bees, as is the case for parasitoid wasps. We then utilize the insights generated to conjecture as to whether or not ecological and anatomical adaptations are reflected in the patterns of venom gene evolution. Throughout the paper, we distinguish between “venom proteins” (or the genes that encode them) and “toxins” (or toxin-encoding genes). The former are those proteins *associated* with the venom system (often secreted in the venom itself) but not necessarily having toxic functions themselves*—*we reserve the designation “toxin” for those gene products with *characterized toxic functions within venom*. Given a permissive definition of the label “venomous” (see discussion), our results suggest that the entire extant Hymenoptera lineage may be descended from a “common venomous ancestor”.

## Results and discussion

### The most prevalent bee venom proteins and their genomic framework

**Fig. 1:**
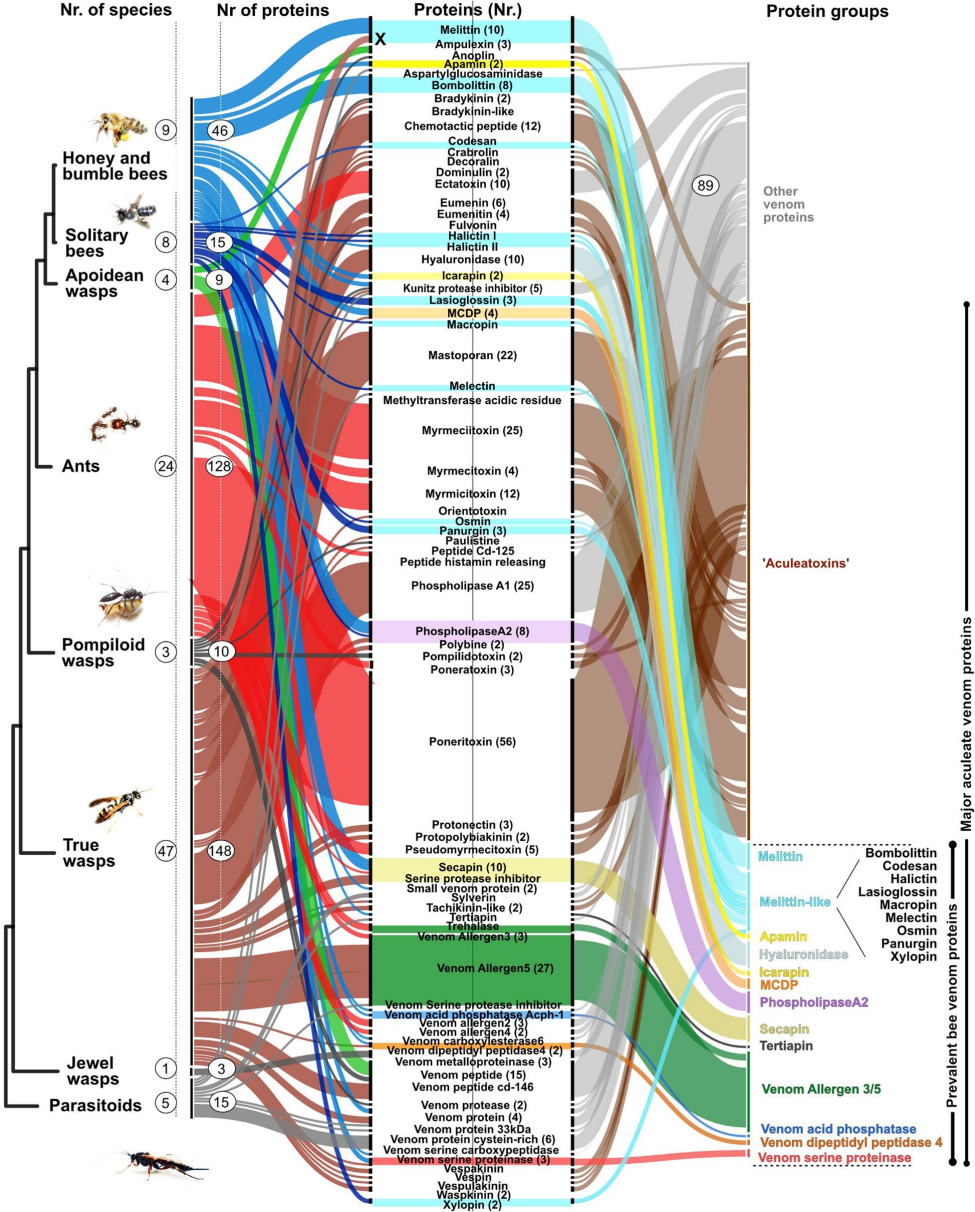
Reviewed venom proteins for hymenopteran taxa in respect to protein and species numbers from UniProt. Major hymenopteran clades are shown on the left (species numbers in circles). The second numbers in circles within the colour-coded lines indicate venom proteins (grouped according to their names). The twelve herein proposed prevalent bee venom protein families (PBVP) are illustrated on the right, together with the toxins proposed as “Aculeatoxins” (brown) according to Robinson et al. [29]. Novel, and further undescribed peptides and proteins are shown in grey. The hymenopteran groups are based on the recent phylogeny according to Peters et al. [33]. Please note that three melittin sequences from wasps are falsely annotated in UniProt as wasp melittins (marked by a black X). Our analyses clearly show that genes encoding peptides highly similar to honey bee melittin are not present in wasps, see also von Reumont et al. [22]. The phylogeny is pruned to the groups for which data is available based on Peters et al. [33]

We establish here a set of 12 proteins that we identify as the most prevalent injected bee venom components based on mining of published sequences, data of toxins with known activity [19, 27, 32] (see Fig. 1), and new, own proteo-transcriptome data.

**Fig. 2:**
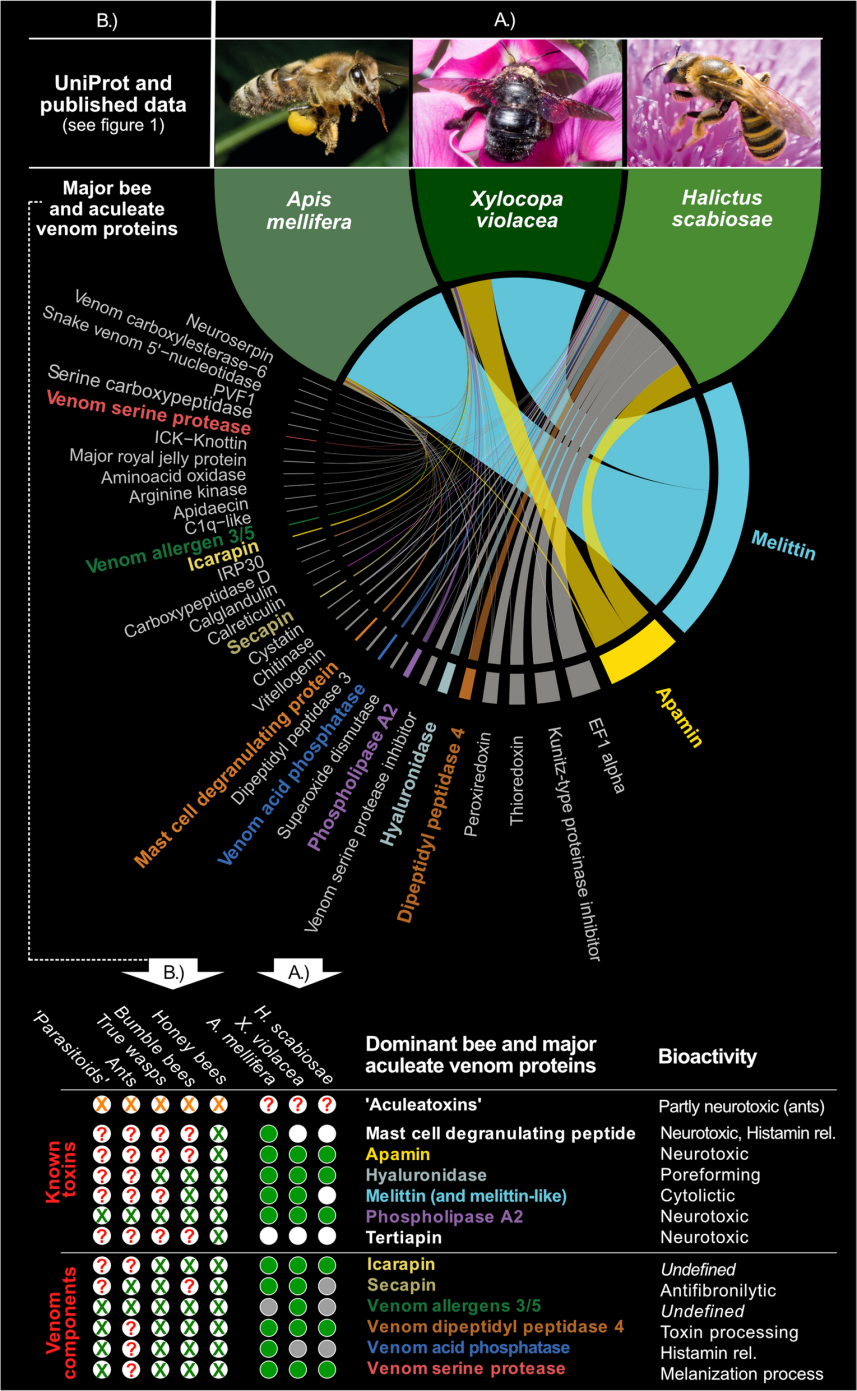
The most prevalent bee venom proteins. Components selected from our own data (A.) *A. mellifera*, *H. scabiosae* and *X. violacea* profiles, and (B.) published bee and aculeate venom components. In (A.) only venom protein transcripts validated by the proteome data are listed. Transcript expression is shown as thickness of the Circos plot lines and based on the percentage of scaled transcript per million (TPM) values including only proteome-validated sequences. The twelve selected venom proteins that we discuss herein further as dominant bee venom proteins are printed in bold in the colour code used for these proteins in this manuscript. Peptide names in white were not identified by our proteo-transcriptome data but are present in published data. For our new proteo-transcriptome data (A.), the green circles indicate venom proteins identified by proteo-transcriptomics, grey circles indicate transcriptome-only hits. White circles illustrate missing data. For published data the green X indicate major components identified in literature, red questions marks highlight missing/unclear data. Orange X highlight the “aculeatoxin” peptides (According to Robison et al. [29], melittin is also a member of the proposed aculeatoxin family, which is separately shown as part of the PBVPs)

New venom profiles were generated for two phylogenetically distant solitary bees, the great-banded furrow-bee (*Halictus scabiosae*), and the violet carpenter bee (*Xylocpopa violacea*). Additionally, we added one eusocial bee, the honeybee (*A. mellifera*), as complementary data (Fig. 2 and Additional files 1, 2, 3, 4, 5 and 6). All three venoms predominantly contained low-molecular-weight peptides, in particular melittin, apamin and mast cell degranulating peptide (MCDP). Larger proteins such as phospholipase A2, venom acid phosphatase, venom dipeptidyl peptidase 4 and venom allergens made up less than 10% of the transcripts based on expression values (see Fig. 2 and “ Methods”). We have to state critically that our heterogeneous picture of venom expression (Fig. 2A) could be reasoned by the difficulty to synchronize the physiological state of venom glands, especially for solitary bees. The transcriptome for *H. scabiosae* was of lower quality compared to the other two species (see “ Methods” and Additional file 7). Given that the focus here is on comparison across the broader clade, we will discuss the species-specific venom composition differences elsewhere, see for example von Reumont et al. [22]. In general, the new profiles corroborate our selection of prevalent bee venom proteins (Fig. 2). Further analysis is restricted to these 12, which include toxins and six auxiliary venom peptide and protein families mostly with known function, but also including two prevalent venom protein families of currently unknown function (Venom allergen 3/5 and Icarapin), see Additional file 8. We refer to these venom components from here on as prevalent bee venom proteins (PBVP).

Two major groups are distinguishable in the PBVP—toxins with characterized acutely toxic functions such as neurotoxicity (e.g. Apamin) or cytotoxicity (e.g. Mellitin), and proteins consistently present in the crude venom presumably as accessory components (Fig. 2). To uncover the evolutionary history of the prevalent bee venom proteins, we analysed corresponding genomic regions by searching for homologues in 29 published genomes (see Additional file 9) of bees and outgroups (sawflies, jewel wasp, ants, paper wasps) and our three genomes of two sweat bees and the violet carpenter bee (See “ Methods” for further details and Additional file 10). The selected taxa span 300 million years of evolution and include representatives of the phytophagous sawflies (Symphyta), the basally divergent hymenopteran lineage. We used the well-annotated *A. mellifera* reference genome to trace venom genes and their flanking genes based on exon regions. We identified orthologs for each exon in other genomes, which were collected into an extended database. We searched all genomes using this database and then manually inspected the results before we inferred the phylogeny of each protein family to establish completeness and microsynteny. “Synteny” refers to shared patterns of gene arrangement (“colinearity”) in homologous genomic regions across taxa which reflect the arrangement and position of flanking exons of genes around venom protein genes (see details in “ Methods”). When sufficiently high-quality genomic sequences are available and genes of interest are located in stable regions, the ability to utilize microsyntenic analyses—comparisons of synteny/colinearity in short stretches of the genome—is a key advantage of comparative genomics. Where sequencing is sufficiently contiguous, these analyses reveal the arrangement of genes and their neighbours as physically instantiated in a chromosomal region. By mapping such regions including genes of interest and their neighbours, it is possible to catalogue rearrangements that occur in diverse taxa.

Put simply, observation of the spatial relations between genes of interest and their neighbours (both complete genes and gene fragments) in one species, enables identification of homologous genes in additional taxa by examination of the sequences that flank these genes. This “genomic context” allows a clearer identification of orthologs than phylogenetic analyses based on proteo-transcriptomics alone and provides insight into the mechanisms of duplication and regulation operative within gene families [8, 34]. Our results indicate that PBVP, including enzymatic components, are present as multi- or single-copy genes in genomic regions stable enough to facilitate comparative microsyntenic analyses. The stability of these regions across investigated taxa suggests that the origins of these genes are ancient, probably occurring in the most recent common ancestor of sawflies, parasitic wasps, and aculeate wasps. Exceptions to this pattern are the short, single-copy genes encoding toxic peptides known from bees such as apamin/MCDP/tertiapin, and melittin, which appear unique to bees or honeybees, indicating much more recent origins.

### Machine learning reveals protein space complexity of hymenopteran venoms

**Fig. 3:**
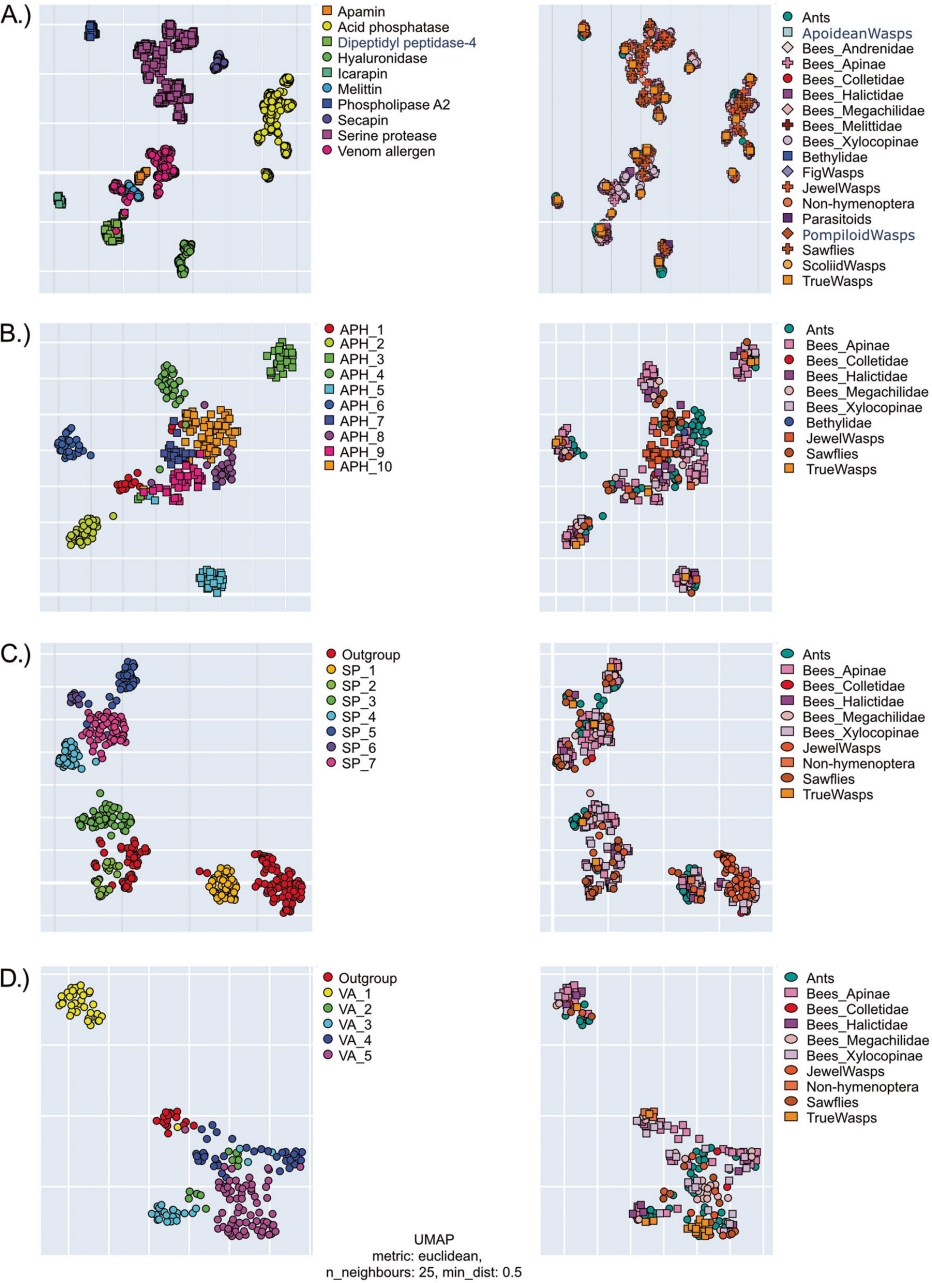
Machine learning generated protein space representations of hymenopteran venoms corresponds with gene phylogeny-based clustering. In each case, the left panel shows the breakdown of subgroups revealed in this study (protein families for the entire dataset, subfamilies for each of the protein families) while the right panel shows the same space coloured by taxa, clearly highlighting that each protein group is a gene clade, not a species clade. **A** Representation of the entire non-redundant dataset. **B** Acid phosphatase family. **C** Serine protease family. **D** Venom allergens family

To address the complexity of the venoms and gain alignment-independent evidence for multigenic families within them, we used a novel approach based on protein language models (Fig. 3). These analyses generate a model of the relations of proteins to each other in a multidimensional “protein space” similar to the concept of a “configuration space” in physics, or an “arbitrary space” in multi-scale cognition [35]. Our protein space incorporates data concerning the structure and function of mature proteins to generate a multidimensional model of protein relations. By observing the clustering patterns of proteins within this space, we can infer their evolutionary relations to one another. In a nutshell (see “ Methods” for the details), a model trained on millions of protein sequences constructs a 1024-dimensional representation of the target data, where Euclidian distances between each of the sequences’ representations (called “protein embeddings”) indicate differences in structure and function. To make those results amenable to visualization, this hyperdimensional space is compressed into 3D (and 2D for figures, but please see Additional file 11 for interactive 3D plots). Of course, such a simplification inevitably leads to decreased representation—PCA compression (see Additional file 11), which is comparable to UMAP used here, retains only 40–50% of the original information. However, even simplified representation still illustrates each of the protein families as standalone groups (with the notable exception of venom allergens, likely because of their divergent functions), and separates most subfamilies within each family.

### Abundant venom proteins are encoded by more widespread single-copy genes

**Fig. 4:**
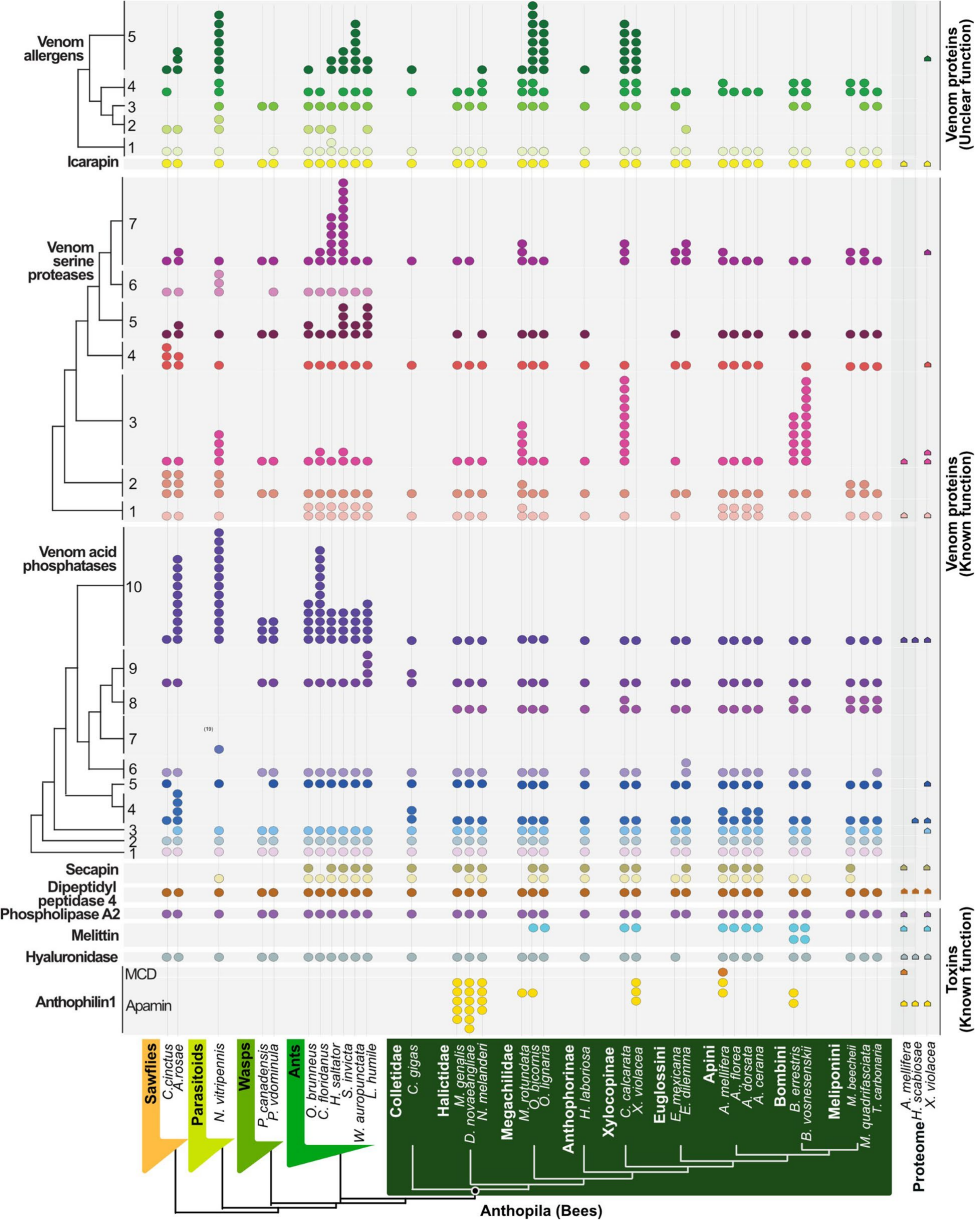
Overview of prevalent bee venom genes. The presence of venom gene orthologs and copy number variation is mapped onto the phylogenetic relationship between the species we surveyed according to Peters et al. [33]. Coloured circles represent genes with identical microsynteny in the genomes of the surveyed species. Please note that tertiapin is now included within anthophilin1 as variant of apamin

Phospholipase A2, hyaluronidase and icarapin are among the most abundant bee venom components [5, 18, 27]. Phospholipase A2 and icarapin are encoded by four-exon single-copy genes, whereas the hyaluronidase single-copy gene features nine exons. Dipeptidyl peptidase-4 has a strongly conserved single gene, which was present in all hymenopterans in our dataset, probably due to its enzymatic role in the maturation of some toxins. These protein families were highly conserved and ubiquitously present in the genomes of bees, wasps and ants (Fig. 4, see also Additional files 10, 11, 12, 13, 14, 15, 16, 17, 18 and 19 for phylogenetic alignments and trees). Our results support the hypothesis that these genes were recruited into venom functions without any associated duplication—similar to co-option of single-copy genes proposed as the main process of venom protein evolution in *Nasonia* [36]. In comparison, phospholipase A2 genes in viperid snakes had multiplied and diversified before recruitment into the venom system [37, 38].

### Some venom proteins form multi-copy gene families with ancient duplication events

Larger duplication and diversification events appear restricted to families of enzymatic or larger proteins and not toxin peptides or proteins. Three venom protein classes in the PBVP showed copy number variation across the dataset: venom allergens 3/5, venom acid phosphatases (APHs), and venom serine proteases (VSP), see Fig. 4. These genes were in stable genomic regions allowing for the tracing of homologous regions between species by screening for microsynteny.

Among the 10 subfamilies of venom acid phosphatases (Fig. 4), the largest expansion of genes occurred in subfamily 7, found exclusively in parasitoid wasps. This may support the hypothesis that ancestral APHs functioned as pre-digestion factors that allowed the offspring of parasitoid wasps to feed more easily on their host [39]. In contrast, gene expansion in subfamily 10 appears to be an ancient pattern found in sawflies (9 genes) and parasitoid wasps (13 genes). In all remaining hymenopterans, only one or occasionally two to three genes are present. A similar pattern was observed for subfamily 5 with ant species having 2–4 copies, while all other hymenopterans (with the exception of *Athalia*) have 1. Subfamily 3 seems to have undergone multiple duplication events in some bee species with up to 10 copies in *Ceratina* and *Bombini*, while other species have 1–2 copies or lost all genes (*Meliponini*), see Additional files 20 and 21 for phylogenetic alignment and tree. In bees, the retained APHs may be adapted to defensive functions, a conjecture potentially supported by the origin of APH subfamily 8, which is unique to bees.

Our analyses divided venom serine proteases (VSPs) into seven subfamilies. Subfamily 7 is represented by 1–4 genes in all hymenopterans but has expanded in ants (10 genes). All seven subfamilies are present in the basal lineages of sawflies and parasitoid wasps, with more diversification in families 2, 3 and 4. In bees, subfamily 6 appears to have been lost (Fig. 4; see Additional files 22 and 23 for phylogenetic alignment and tree). VSPs are dual function toxins in bees, triggering the phenoloxidase cascade leading to melanization when injected into insects but acting as spreading factors when injected into mammals, similar to snake VSPs with fibrinogen-degrading activity [40]. We hypothesize that the expansion of VSP genes may be linked to this dual function, achieving more effective defense against insects, arthropods and mammals.

Venom allergens 3/5 have been identified in many hymenopterans [5, 41] and we distinguished five subfamilies in our study. Subfamily 5 appears to have undergone greater diversification in sawflies, parasitoid wasps, ants and solitary bees (*Ceratina*, *Osmia*). Only a single member of subfamily 5 is present in the solitary bees *Habropoda*, *Colletes* and *Nomia.* Eusocial wasps and bees of the family Apidae (*Apis*, *Bombus, Melipona*, *Frieseomelitta* and *Eufriesea*) appear to have lost all subfamily 5 genes. Subfamily 1 is present only in parasitoid wasps and ants with a single gene in *Euglossa*. Other subfamilies generally have a single copy in every species with subfamily 4 occasionally experiencing duplication. In general, the distribution of genes in the venom allergen family is dynamic but shows some phylogenetic patterns (see Additional files 24 and 25 for phylogenetic alignment and tree).

Two secapin genes were present in most genomes but were absent in sawflies (indicating an origin in the stem Apocrita) and wasps of the genus *Polistes*. This class of peptides displayed N-terminal sequence variation but strong C-terminal conservation (see Additional files 26 and 27 for phylogenetic alignment and tree). The location of both genes was also strongly conserved, with one always present between exons of the neurexin-1 gene and the other located near the carbonic anhydrase-related protein 10. Our inability to locate both genes in some species may reflect technical issues relating to genome quality and/or the more general challenges associated with the location of small and highly variable genes.

### Apamin is restricted to honeybees and is part of the larger bee-unique toxin family Anthophilin1

**Fig. 5:**
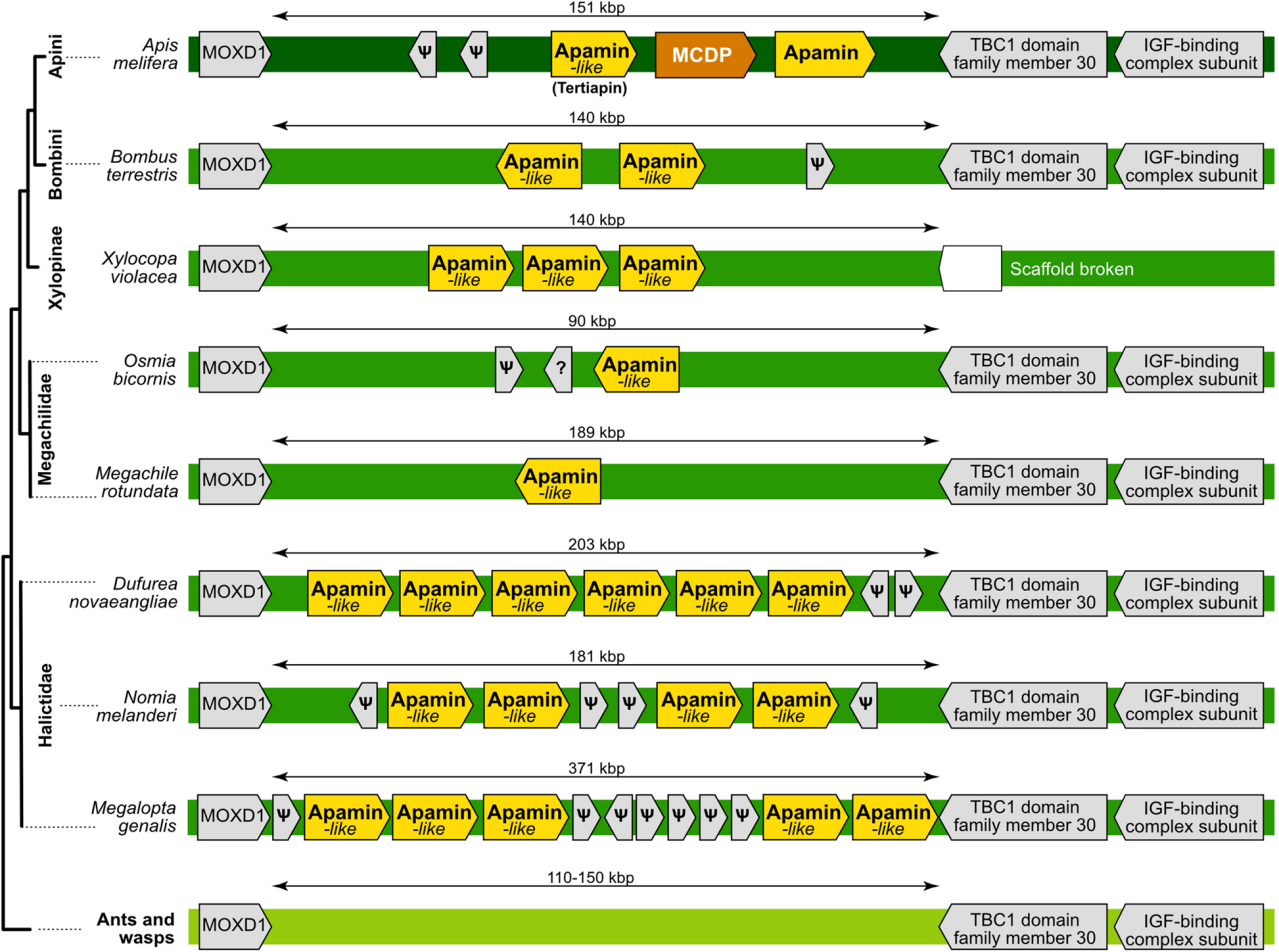
Microsyntenic pattern for the apamin family (Anthophilin1). Question marks indicate coding sequences with products of unknown functions. Pseudogenes are symbolized by ψ. The arrows reflect gene orientation. We show here only species for which the genomic sequence in the region with apamin genes is contiguous. Note that “apamin-like” genes are also known as “tertiapin”. *Apis* lineages are in dark green, other non-Apini bees in grass green and ants and wasps in light green

Apamin, a dominant *A. mellifera* venom component, is encoded by a three-exon gene located next to a very similar three-exon gene encoding MCDP. This tandem duplication is flanked by MOXD1 homologue 2 and TBC1 domain family member 30. Although the two flanking genes are present and identically arranged in the genomes of all the bees we surveyed, we did not detect the full set of apamin or MCDP exons outside of the genus Apis (Fig. 5). Genomic analysis confirmed that apamin and MCDP (from *Apis*) are restricted to the Apini clade (*Apis* spp.). In addition, we identified a novel apamin-like gene locus in *Apis mellifera* located right next to MCDP gene—400 bp upstream. This gene encodes the described honeybee toxin peptide named tertiapin [42]. Multiple uncharacterized genes that share microsyntenic position and intron–exon structure with this apamin-homologue (Tertiapin) were observed in Bombini and some other non-*Apis* bees. These apamin-like genes encode peptides that share the cysteine scaffold and signal peptide structure of apamin, MCDP and tertiapin. They were widespread in bee genomes and we identified six copies in the *Dufourea* genome, five in *Nomia* and *Megachile*, two in *B. terrestris* and a single copy in *Osmia bicornis*, *Habropoda* and *Megachile*. This pattern may be indicative of the derivation of apamin and MCDP from the more widespread tertiapin. We identified no similar genes or exons of apamin in homologous regions from other hymenopterans or in other parts of their genomes.

The apamin-like sequences, we discovered in the core venom profile of *Xylocopa* and *Halictus* indicate that apamin and MCDP are members of a variable bee-unique family of apamin-like peptides that undergoes independent duplication events in different lineages. We propose here to name this novel family Anthophilin1, reflecting its uniqueness to several lineages within bees (Anthophila), see Additional files 28 and 29 for phylogenetic alignment and tree.

### Melittin is restricted to the bee lineage

**Fig. 6:**
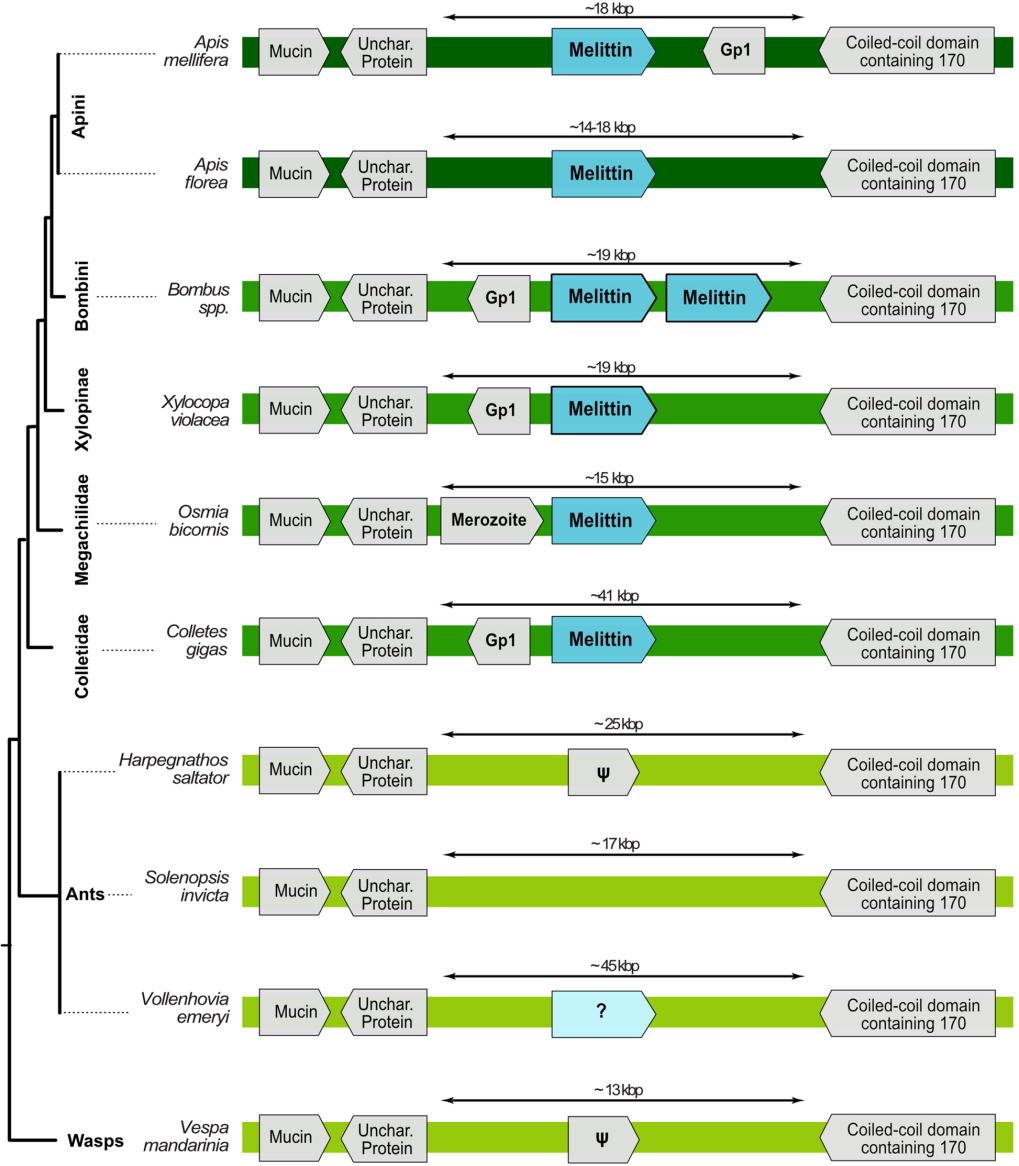
Microsynteny around the melittin sequence. All species for which the genome data allowed for microsyntenic analysis are shown. *Vollenhovia emeryi* was not included in other genomic analyses due to its relatively low genome quality. However, it is shown because it was the only one of the eight analysed ant species that features a seemingly related gene in the correct position but with a very different mature sequence. Genes labelled with ψ in ants and wasps bear little similarity with melittin genes; however, they might be sister genes to the melittin group that underwent severe pseudogenization. Note that *Osmia* melittin is also called “osmin”, *Colletes—*collectin, *Bombus*—bombolittin, and *Xylocopa*—xylopin. *Apis* lineages are in dark green, other non-Apini bees in grass green and ants and wasps in light green

Melittin is a pain-inducing peptide in *A. mellifera* venom [27, 43]. The synteny of the *A. mellifera* genome shows that melittin is encoded by a two-exon single-copy gene located between two four-exon genes, one of which encodes *vegetative cell wall protein gp1* while the other remains uncharacterized. Melittin-like sequences in other *Apis* species (*A. dorsata*, *A. cerana* and *A. florea*) feature similar microsynteny (Fig. 6). Other bee species also possess melittin-like sequences (bombolittin, osmin, collectin, lasioglossin, melectin, codesane, halictin and macropin) [44–48]. Microsynteny analysis provided evidence that osmin, collectin, bombolittin and xylopin are orthologous in at least some species from the genera *Colletes, Osmia* and *Bombus* (Fig. 6).

In *Bombus vosnesenski*, the melittin gene has undergone a tandem duplication that is apparently unique to *Bombus*. Some *Bombus* genomes show assembly gaps in this region, preventing the detection of all exons, but recently published genomes of several *Bombus* species [49] show the same sequence and duplication pattern in the microsyntenic region identified in *B. vosnesenski* (Fig. 6). Although tracing the corresponding genomic region in non-bee Aculeata proved to be difficult because of its relative instability (low synteny/colinearity), we successfully located it in ants and wasps, which lacked melittin homologues (both mucin and coiled-coil domain containing 170 genes were present in the same orientation as in bees). However, one ant genome—*Vollenhovia emeryi* (excluded from our main genomic analysis due to the relatively low genome contiguity)—had a superficially similar looking gene in almost the exact location (Fig. 6). That gene has a proline-rich propeptide resembling that of melittin; nevertheless, its mature form is very different. The protein is 39 aa longer than *A. mellifera* melittin (109 vs 70) and only 22 out of 70 residues are shared between them (see Additional file 30). We conclude here that our results support the hypothesis that melittin is restricted to bee lineages; however, its ancestral gene might have had homologues in ancestors of wasps and ants, see Additional files 31 and 32 for phylogenetic alignment and tree.

### Gene synteny and assisting machine learning model of “protein space” cast doubt on aculeatoxin hypothesis

**Fig. 7:**
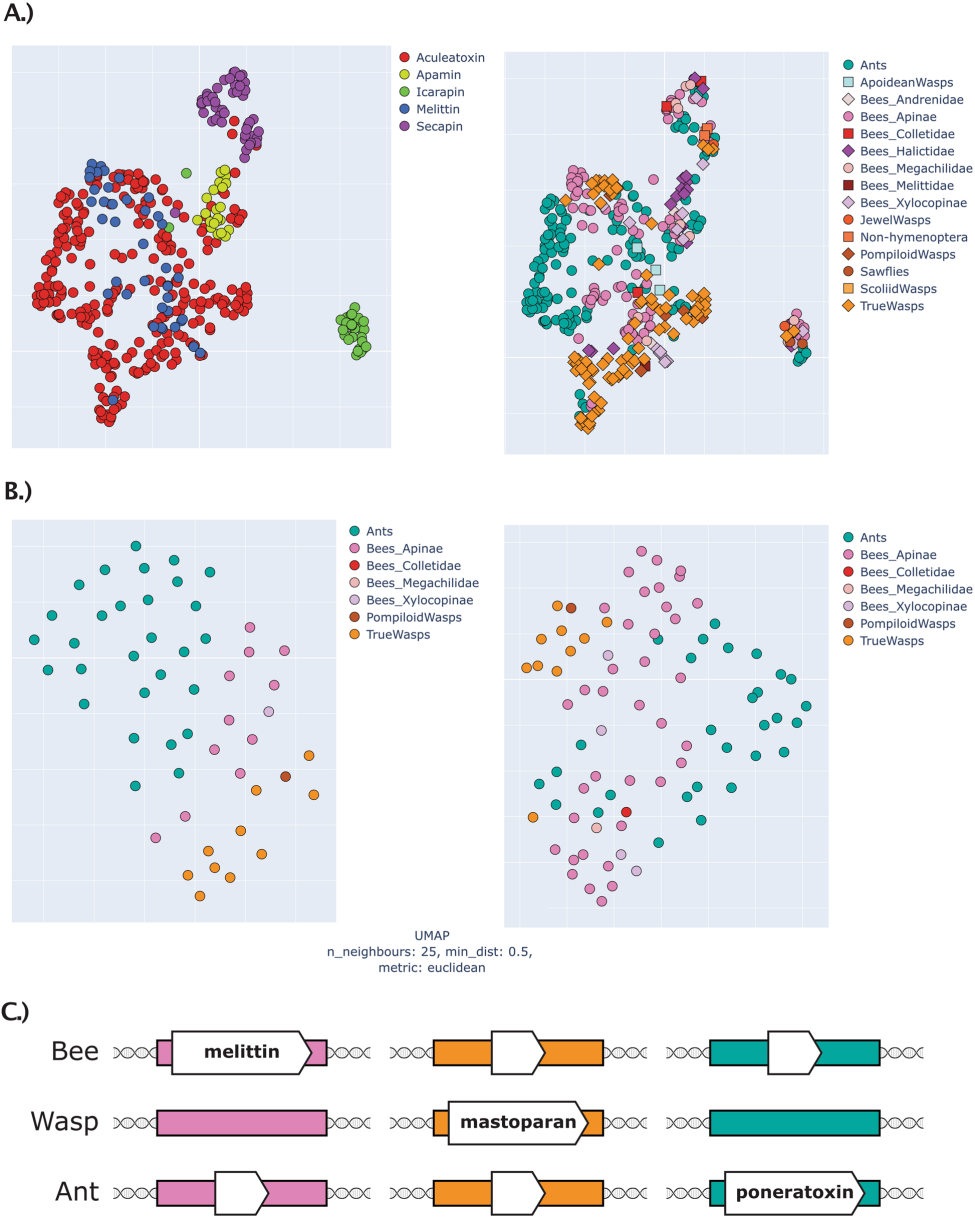
“Protein space” of small peptidic aculeatan toxins as revealed by machine learning analysis and their genomic position in respect to each other. **A** Combined data of available verified toxin sequences from Robinson et al., and the present study (including ToxProt part of the UniProt) for all peptidic toxins, on the left coloured by protein family, on the right—coloured by taxa. **B** Data from Robinson et al., on the left sequences with signal peptide included, on the right only mature peptides. For the interactive plots see Additional file 11. **C** Schematic of genomic position of the three groups of hymenopteran toxins. Coloured rectangles represent regions of microsynteny: pink for melittin, orange for mastoparan and green for poneratoxins. See text for details

Given the short peptide sequences and consequent challenges for phylogenetic analyses, we utilized a novel, alignment-independent machine learning approach (see “ Methods” section for the detail) to test the proposition of aculeatoxins by Robinson et al. [29]. We focused on small peptidic aculeate toxins, but especially on melittin, which is according to Robinson et al. based on signal and propeptides, a member of the “aculeatoxin” family which origins with aculeates (see Fig. 1). We used all sequences that Robinson et al. [29] presented in their study (kindly provided to us by the authors) and all melittin-like toxins known from bees that are included in our study. We created three datasets: (panel A on Fig. 7) mature sequences from Robinson et al. data, all unique hymenoptera venom peptides from this study and ToxProt as part of UniProt (see Additional file 33); (panel B on Fig. 7) Robinson et al. data with (Additional file 34) and without (Additional file 35) signal/propeptides—the latter dataset includes more sequences since bee melittins are mostly known from proteomic studies and therefore only their mature sequence is known. When comparing proposed aculeatoxins with other hymenopteran peptides, it becomes apparent how the sequence space occupied by this hypothetical protein family bleeds into that occupied by apamins and icarapins, and also exhibits a very clear separation by taxa (in contrast to established protein families from Fig. 3). Sequences from bees, wasps and ants do not intermix, which is even more apparent on the smaller scale seen in Fig. 7 panel B. It is possible that different ecological pressures separated ants, wasps and bees’ peptides to such an extent that they occupy distinct areas of structure–function space; however, together with the synteny (see below), this analysis finds no support for the aculeatoxin hypothesis (Fig. 7). On the other hand, the results reveal a close similarity between bee and wasp peptides, which was even more apparent when signal peptides were removed (in contrast to the reasoning of Robinson et al., which is based on similarity among signal and propeptides alone).

Despite our focus on the evolution of bee venom genes, we also searched our synteny analyses for representative toxin peptides of wasps and ants to trace their possible occurrence in bees. From vespid wasps, we included mastoparan, eumenine and vespakinin, and from ants poneratoxins and myrmecitoxins. We did not find any genomic location and sequences that match eumenine, vespakinins and myrmecitoxin genes in any of our bee data and thus did not include them in further analyses.

However, we located poneratoxin-like sequences near or within the atrial natriuretic peptide-converting enzyme in all ant genomes of our dataset. The number of the genes seems to vary between the species. Although the genomic region appears unstable, we located atrial natriuretic peptide-converting enzymes and some of its nearby and proximal neighbouring genes in *Vespa* and *Apis*. No genomic feature in the genome of *Vespa* seems to relate to poneratoxins, while *Apis* features an uncharacterized small 2 exon gene in a similar position. Aligning it with poneratoxins from *H. saltator*, *S. invicta* and *O. brunneus* (extracted from genomic regions) revealed its close similarity to the 5′ end of prepro mRNA of the longest of *H. saltator* poneratoxins. This finding indicates that the original gene got truncated and only its 5′ part is retained in *Apis*.

We restricted our synteny analysis for mastoparan to the main representative species of bees, ants and wasps and substituted *Polistes* spp. with *Vespa mandarinia* because in both *Polistes* assemblies the mastoparan homologues are located on small scaffolds that prevent the tracing of the synteny. We located mastoparan in the genome of *Vespa mandarinia* and used it as an anchor. The gene is located downstream from LIM/homeobox protein Lhx9-like. The genomic region appears stable and we were able to locate it in Apis mellifera and *Solenopsis invicta*. However, both species only show annotated pseudogenes (no reading frame seems to resolve in a functional product) in this genomic region roughly in the expected position of mastoparan. Both pseudogenes have three exons and in both cases one of the reading frames encodes for a proline-rich (pr) region that could have resembled the mastoparan’s propeptide before the pseudogenization.

Thus, microsyntenic analyses reveal that melittin, mastoparans and poneratoxins are non-homologous and reside in different genomic regions (Fig. 7, panel C); however, the protein space analyses may reveal evidence of convergence. Taken together, the results of these analyses indicate that melittin is likely unique to bees but gravitates towards mastoparans (in particular) and poneratoxins (to a lesser extent) in protein space, possibly because of functional convergence. It is important to note that all members of the proposed aculeatoxins are processed by the same enzyme, DPP4. This common processing may contribute to similarities in signal and propeptide sequences, which is the key aspect of the aculeatoxin hypothesis.

If we assume that this shared pattern of proline/glutamic acid-rich sequences in the propeptide region and some parts of signal peptide is an apomorphy of a gene family, and that mastoparan, melittin and “poneratoxins” are members of that family, then it is extremely likely that this gene family predates Aculeata. Because all three clades (ants, bees and wasps) seem to possess genes that relate to all three (sub)groups, it suggests that this proposed clade of genes existed as a group of genes, and not as an individual ancestral gene, before the split of Aculeata. Moreover, this group of genes is quite likely then to be related to some of the numerous hymenopteran toxins, some of which (like *Bracon* peptidic toxins) exhibit the pr motif. The richness and evolvability of these “hymenopteratoxins” is exceptional.

### Gene expansions are restricted to few venom protein families in major taxa

Most PBVP are encoded as single-copy genes (Fig. 3), indicative of single gene co-option. Our data supports the hypothesis that gene duplications are a less prevalent evolutionary mechanism in the evolution of hymenopteran venom components than has been shown for (e.g.) snakes. This pattern was previously observed in parasitoid wasps (*Nasonia*) [36]. However, our results indicate a more distinct pattern in which heavier protein and enzyme components represent those families of venom proteins in which large gene duplications and expansions have occurred in conserved genomic regions. These expansions are restricted to particular subfamilies and larger hymenopteran clades (Fig. 3). The gene duplications and subsequent gene expansions of venom serine proteases, venom allergens and venom acid phosphatases appear to be “simple” events restricted to the expansion of few genes. This is in contrast to other venomous organisms that have been studied more extensively, such as snakes and cone snails, in which venom genes have evolved rapidly by extensive multiplication, expansion and subsequent deletion [12, 50–53]. It should be noted, however, that this picture is based on our preselected PBVP, which includes the most common venom components described.

Venoms are secretions which primarily function (when “actively delivered” via bites or stings) to deter or subdue target organisms. Venoms contain a variety of molecules and not all are necessarily associated with the primary function of the secretion. Some are of as yet unknown function, or may be epiphenomenal (i.e. present in venoms for contingent reasons not associated with any particular functional role). Our results indicate that genes encoding (characterized) toxins and those encoding other (associated) venom proteins (often only identified by proteo-transcriptomics without any further functional characterization) evolve differently in bees, suggesting a genuine functional distinction between these groups. This finding should be tested further in the future using extended venom profiles. Complementary activity studies are important to address the still undefined biological functions of many venom components, for example venom “allergens”, which would in turn support a better interpretation of evolutionary patterns. Venom allergens (3/5) show a more heterogeneous pattern of gene duplications than other gene families, especially in subfamily 5. This subfamily has expanded in parasitoid wasps, leafcutter bees (Megachilidae) and carpenter bees (Xylocopinae), but has been lost in other Apidae lineages. We can only speculate about the original and actual biological function of venom allergens in general because until today the only *activities* characterized are related to immune responses in mice and humans linked to allergic reactions [54]. No study so far has addressed the possible bioactivity linked to the ancestral and venom variant’s biological *function*. However, the strong allergenic activity may reflect an ancestral immunomodulatory function in sawflies linked to the modulation of the immune response of plants, which was later adapted to animal hosts in more derived aculeate lineages.

### Bee-specific toxin genes encoding for short peptides

Bees produce apamin and melittin as predominant venom components [27], but their genomic origin beyond the honeybee lineage has not been investigated before. One major difference between these toxin peptides and previously discussed venom components is that the genomic region in which they are encoded appears more dynamic. This picture is also reflected by taxon-restricted gene duplications. The genomic region containing a tandem repeat of apamin and mast cell degranulating peptide in *Apis* was identifiable in other bee genomes based on microsynteny and the characteristic cysteine scaffold. Interestingly, we discovered multiple duplication events each restricted to single bee lineages. Our conclusion based on this pattern is that apamin and mast cell degranulating peptide are members of a so far unrecognized, highly variable bee-unique peptide family, which we named Anthophilin1. The genes of this family seem to diversify independently in different bee lineages. Interesting is that in snakes and sea anemones the expansion of toxin gene families is shown to be linked to their selection to generate larger quantities of the venom than novel function [13, 15]. Whether the duplication events are linked to neofunctionalization or co-option (as one dominant venom component) remains to be addressed in future studies, the scenarios of gene duplication in venom evolution can be more complex than they often appear [8]. These should include more contiguous genomic data from additional bee lineages and complementary venom proteomes to better understand the recruitment and diversification processes of members of this family in bee venom.

We identified melittin in a genomic region with conserved synteny in the genera *Apis, Osmia, Ceratina* and *Bombus* (families Megachilidae and Apidae), with a tandem duplication in bumblebees. Synteny confirmed that melittin-like peptides produced by solitary bees are members of the melittin family. Accordingly, melittin is not unique to *Apis* but originated before the divergence of megachilid and apid bees. We did not find a syntenic region or sequences similar to melittin in genomes of bees from the families Andrenidae, Halictidae and Colletidae. Whether or not melittin evolved in earlier bee lineages and underwent secondary loss in some families remains unclear from our data due to the lack of high-quality genome assemblies for the early-diverging bee lineages. Our data further indicates that the ant *Vollenhovia* possesses a gene which may be distantly related to melittin; however, the mature sequence looks very different—it is 29 amino acids longer and only 22 aligned residues are shared. Nevertheless, we cannot rule out the possible origin of melittin in earlier aculeate lineages until a larger sampling of taxa from these and earlier bee lineages are available with high-quality proteo-transcriptome-genome data. Regardless, our data suggests that melittin is co-opted as a single-copy gene as one major component in bees. In future studies, this hypothesis should be further tested by analysing more proteo-transcriptomic venom profiles linked to genomic data.

### Most bee core venom proteins originated in early hymenopterans

**Fig. 8:**
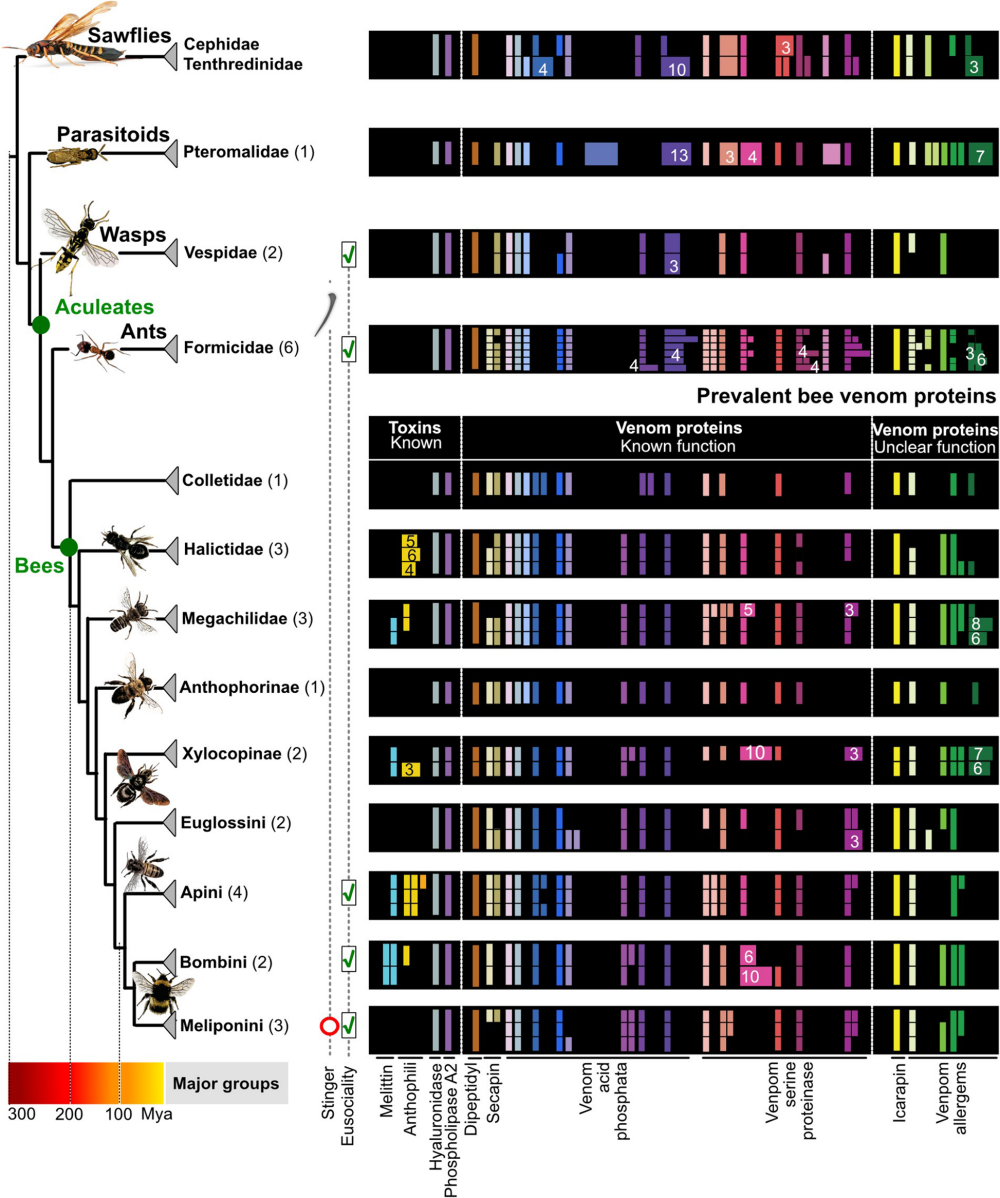
Simplified visualization of the prevalent bee venom proteins and their representation in outgroup taxa. The numbers of genomes are shown in brackets after the family names. Genes are colour-coded and feature a colour range for duplicates. Duplications are summarized by numbers. Phylogeny and divergence times are shown as previously described in Peters et al. [33]. The nodes for monophyletic aculeates and bees are highlighted in green. The red lined circle indicates the secondary loss of the stinger in sweat bees (Meliponini)

The pattern we infer reveals an ancient origin for most of the PBVP in bees (Fig. 8). Most subgroups of major venom protein gene families exhibit clear-cut orthology with genes already present in the earliest hymenopteran lineage (sawflies). Female sawflies use their ovipositor to lay eggs in plants but also co-inject proteins that biochemically interfere with the physiology and immune response of plants to ensure the offspring’s survival, thus resembling an ancestral venom system [4]. The composition of these original hymenopteran venoms has not yet been studied in detail.

Our results suggest that the most prevalent venom genes present in bees today were already present in the early Triassic in ancestors of the symphytan lineage, predating the radiation of apocritans starting more than 200 million years ago (Fig. 8) [33]. The restricted waist of apocritans is needed to manoeuvre the ovipositor in such a way that allows its use for predation, parasitism or defense, and only in aculeate hymenopterans (ants, bees and wasps) is the retractable ovipositor modified into a stinger used exclusively for venom injection. Our data suggest that genes encoding the PBVP emerged before the morphological adaptations of a narrow waist and the stinger in aculeates did, which gave this group its common name—the stinging wasps. The core of the bee venom profile, including known allergens such as phospholipase A2, icarapin and hyaluronidase, was not only already present in sawflies, but is also still present in a group of bees that has secondarily reduced or lost its stinger (stingless bees, Meliponini).

If one accepts Symphyta as “venomous”, based on their injection of molecules that modulate the physiology (particularly the immune system) of target organisms (to facilitate feeding of the next generation, similarly to parasitoid wasps), then one might consider the hymenopteran lineage as “descending from a common venomous ancestor”. Indeed, this might be much less controversial an assertion for this order than it has turned out to be for toxicoferan reptiles (see, e.g. [55] and subsequent discussion in the journal *Toxicon*). In this case, our data is consistent with the idea of continuous evolution (i.e. without sharp distinctions or saltatory events) of the hymenopteran venom system through various changes in associated anatomy and ecology. The core of the venom arsenal, comprised of larger proteins which function as immunomodulators or spreading factors, may have been in place early on. Subsequent evolution focused then on the origin and diversification of lineage-specific arrays of peptides which are tailored to the specific venom function (e.g. defence, parasitism, predation) and target (plants, insects, vertebrates) in each lineage. Thus, while the peptidic toxins are unique to each lineage within the Aculeata (contrary to the aculeatoxin hypothesis), most enzymatic components are broadly shared, albeit with varying degrees of expansion of specific subfamilies. These differential expansions of enzyme-encoding gene families (e.g. serine proteases) may represent the kind of evolutionary tinkering observed in redundant arrays of toxin-encoding genes in other venomous taxa (see, e.g. Jackson et al. [51]), in which slight changes confer adaptation to the biochemical particularities of a new ecological reality. Members of such enzyme classes may thus vary in their activity on specific substrates, linked to modified morphology of the venom apparatus, but are never rendered inactive due to broadly applicable modes of action (i.e. targeting substrates generally conserved across taxa as diverse as plants and vertebrates). The subject of this study, bees, seems to support this view by having little variation in their venom genes, other than within the Anthophilin1 and Melittin groups.

Finally, if we accept the proposition that the unique peptides of ants, wasps (stinging and non-stinging) and bees themselves form a clade, in light of our findings we have to “upgrade” them from “aculeatoxins” to “hymenopteratoxins”. Taken together with the rest of the available knowledge on the hymenopteran venom system, we could then conjecture that these “hymenopteratoxins” (together with DPP4 that processes them) lie at the core of the hymenopteran venom cocktail. Multiple less potent toxins, like phospholipase A2 or trypsin-like proteases, both act as helpers for the function of the main weapon—the peptide, while also adding the effect of general toxicity on their own. A few proteins perform auxiliary functions like increasing membrane permeability (hyaluronidases) or causing allergy (venom allergens, presumably). There is more to the Hymenoptera venom system than this, however, if such a concerted system of enzyme facilitators and a peptide warhead is indeed characteristic of the venom of Hymenoptera, further questions arise: which “toxins” are the more ancient, the peptides or the enzymatic helpers, and what are the homologues of the hymenopteratoxin peptides in other insects (if it is not a case of de novo gene evolution)? The enzymatic helpers have clearer homologues (located in homologous genomic regions, an indication of orthology), though establishing direct orthology with synteny analysis was beyond the scope of this study.

## Conclusions

Our comparative analyses provide insight into the origins and evolution of toxin genes in bees. We found that most genes encoding predominant bee venom proteins originated at the base of the hymenopteran tree, i.e. were potentially present in the “venom” of the last common ancestor of phytophagous sawflies and apocritan Hymenoptera more than 280 million years ago (Fig. 8). Only the short peptides melittin and the (herein newly described) family Anthophilin1, which is constituted by apamin, apamin-like and MCDP-like genes, are unique to bees. Gene duplications occur, but only in certain (not major toxin) protein families and in only a few hymenopteran lineages, reflecting a diverse pattern of gene origin. Our results thus indicate that short peptides and venom protein genes probably evolve under different evolutionary processes. This study of the PBVP demonstrates that the evolution of bee venoms contrasts with evolutionary patterns in other venomous lineages and hence promises several new directions for future comparative studies.

## Methods

### Data mining of hymenopteran venom proteins and genomes

Reviewed venom proteins of hymenopterans were searched in UniProt resulting in 372 protein matches from 101 species (Fig. 1 and Additional file 8). Additionally, we searched publications for sequences that are not provided in UniProt and included finally three bee toxins Halictin I and II from *Halictus sexcintus*, and Codesan from *Colletes daviesanus*. For our comparative genomic analysis of venom toxin proteins across the order Hymenoptera, we made use of 29 publicly available genome sequences given in Additional file 9 and three novel genomes of solitary bees.

### Venom gland RNAseq analyses

For venom gland transcriptomics, 15 individuals of *X. violacea*, 17 individuals of *H. scabiosae* and 15 individuals *of A. mellifera* were collected June–July 2019/2020 in the alluvial area of the River Wieseck in Giessen, Germany, and the beehive at the Institute for Insect Biotechnology at Justus-Liebig-University Giessen (Collection permission HNLUG Giessen IV.2 R28).

Whole venom systems (Glands and reservoir) were dissected and washed on ice under sterile conditions and the tissue was preserved in RNAlater (Thermo Fisher Scientific) for subsequent RNA sequencing. RNA extraction, library preparation and short-read RNA sequencing were outsourced to Macrogen (Seoul, Korea) for *A. mellifera* and *X. violacea*e and to Novogene (Cambridge, UK) for *H. scabiosae*. In short, RNA was extracted with Trizol and the cDNA libraries (150 bp, paired end reads) were sequenced using a low input protocol (Illumina Truseq) on an Illumina HiSeq2500 (Macrogen) and Illumina NovaSeq (Novogene). For *H. scabiosae*, an in-house ultra-low input protocol was used by Novogene due to very low RNA concentration and quantity. All RNASeq raw data of *X. violacea*, *A. mellifera* and *H. scabiosae* were generated within the present study and are accessible in GenBank via the BioProject PRJNA733472 (SRA entries: SRR14690757, SRR14690758, SRR14690759) [56–58]. Venom gland transcriptomes were assembled separately using Oyster River Pipeline v2.2.6 [59]; for resulting BUSCO values, see Additional file 7.

**Fig. 9:**
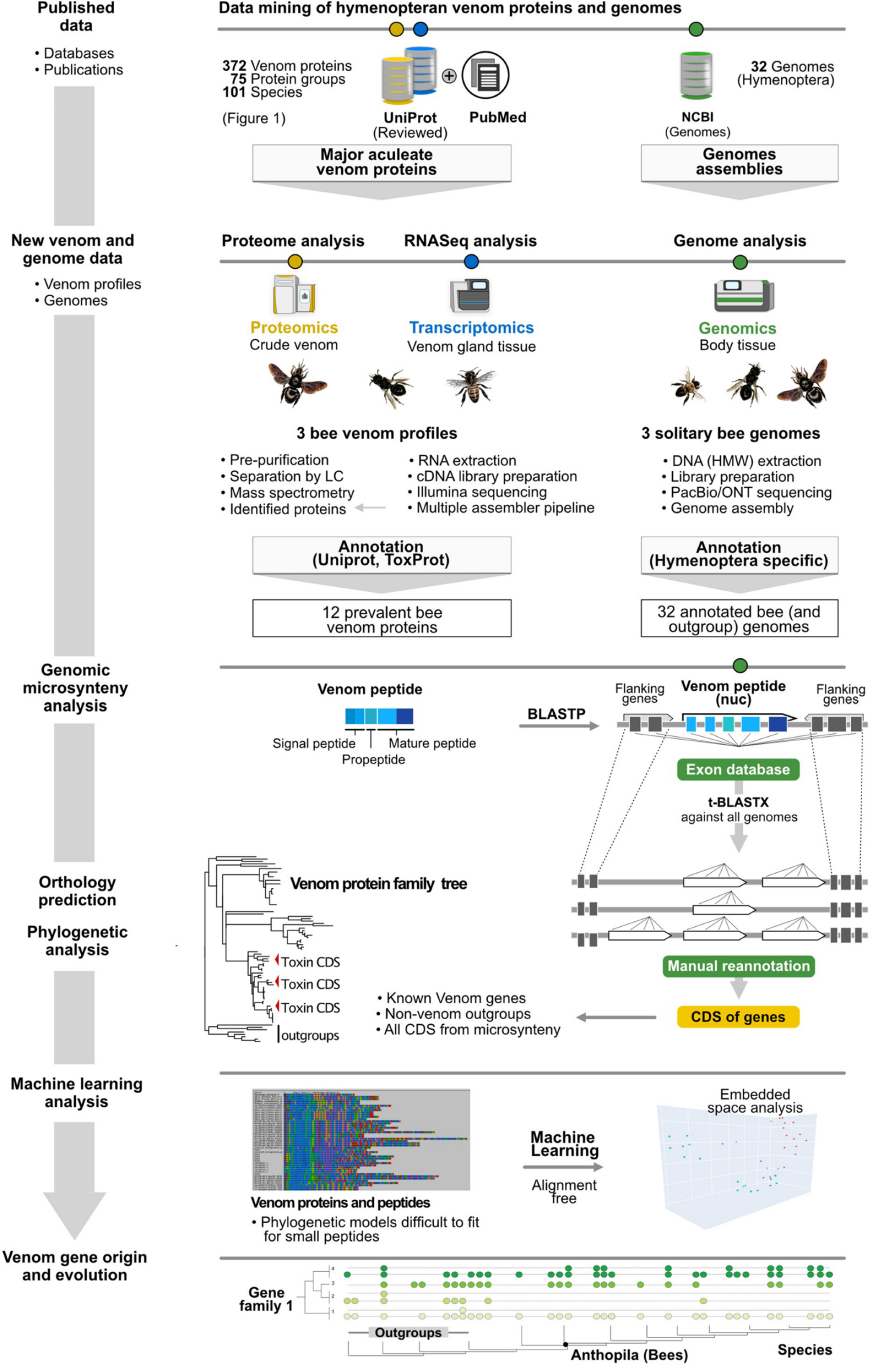
Description of the proteo-transcriptomic and genomic workflow applied in this study. Details of each step are given in material and methods

The resulting assemblies were processed using Transdecoder (minimum length 20 amino acids) to predict peptides, and Kallisto v0.46 [60] to calculate individual transcript abundance, see Additional files 1, 2 and 3. The assembled transcripts and their corresponding longest open reading frames ORFs from Transdecoder were used as local BLAST queries against ToxProt and UniProt (the latter limited to insects only) with an e-value cutoff of 1 × 10^−3^, see Fig. 9. Any highly abundant (TPM > 100) transcripts without significant matches were manually screened using BLAST, InterPro scan and Predict Protein online suites to determine the closest characterized homologue. For subsequent venom protein identification, we only included transcripts identified in our proteomic dataset representing proteins secreted in the venom system. To compare subsequently all venom proteins in the three datasets we calculated the percentage of scaled TPMs using the package txtimport on R, the script is available via github (https://github.com/marivelasque/VenomEvolution.git), see Fig. 2 and Additional files 1, 2 and 3.

### Proteome analysis of crude venom

We extracted crude venom of all specimens from glands and venom reservoirs by squeezing with forceps in sterile ultrapure water (Thermo Fisher Scientific, Waltham, MA, USA) after prewashing twice to minimize hemolymph contamination. All transcriptome assembly-based predicted ORFs were used as specific databases to identify peptides and proteins detected by mass spectrometry from crude venom of the collected specimens. For the tryptic digestion of the crude venom from *H. scabiosae*, we dissolved 10 µg of protein in 10 µl 10 M urea containing 0.1% ProteasMax (Promega, Madison, WI, USA). Cysteine residues were reduced with 5 mM DTT (30 min at 50 °C) and modified with 10 mM iodoacetamide (30 min at 24 °C). The reaction was quenched with an excess of cysteine and trypsin was added at a protein:enzyme ratio of 40:1 in 100 µl 25 mM ammonium bicarbonate (Sigma-Aldrich, Taufkirchen, Germany). After incubation for 16 h at 37 °C, the reaction was stopped by adding 1% trifluoroacetic acid (TFA). The sample was purified using a C18-ZipTip (Merck-Millipore, Darmstadt, Germany), dried under vacuum and redissolved in 10 µl 0.1% TFA. LC–ESI–MS analysis was carried out at 35 °C by loading 1 µg of the sample in 0.1% formic acid (Sigma-Aldrich) onto a 50-cm µPAC C18 column (Pharma Fluidics, Gent, Belgium) mounted on an UltiMate 3000RSLCnano (Thermo Fisher Scientific). Peptides were eluted with a linear gradient of 3–44% acetonitrile over 240 min followed by washing with 72% acetonitrile at a constant flow rate of 300 nl/min. They were then infused via an Advion TriVersa NanoMate (Advion BioSciences, New York, NY, USA) into an Orbitrap Eclipse Tribrid mass spectrometer (Thermo Fisher Scientific) in positive-ionization mode with a NanoMate spray voltage of 1.6 kV and a source temperature of 275 °C. Using data-dependent acquisition mode, the instrument performed full MS scans every 3 s over a mass range of *m/z* 375–1500, with the resolution of the Orbitrap set to 120,000. The RF lens was set to 30%, and auto gain control (AGC) was set to standard with a maximum injection time of 50 ms. In each cycle, the most intense ions (charge states 2–7) above a threshold ion count of 50,000 were selected with an isolation window of 1.6 m*/z* for higher-energy C-trap dissociation at a normalized collision energy of 30%. Fragment ion spectra were acquired in the linear ion trap with the scan rate set to rapid, the mass range to normal and a maximum injection time of 100 ms. After fragmentation, the selected precursor ions were excluded for 15 s for further fragmentation.

Prior to shotgun proteomics, the *X. violacea* and *A. mellifera* venom samples were denatured, reduced, and alkylated. Briefly, each sample (~ 50 μg) was dissolved in 89 μl 100 mM triethylammonium bicarbonate (TEABC), and cysteine residues were reduced by adding 1 μl 1 M DTT (30 min at 60 °C) and modified by adding 10 μl 0.5 M iodoacetamide (incubation for 30 min in the dark). We then added 2 μg trypsin (Promega) in 100 mM TEABC and incubated overnight at 30 °C. The peptides were then purified and concentrated using OMIX Tips C_18_ reversed-phase resin (Agilent Technologies, Santa Clara, CA, USA). The peptides were dehydrated in a vacuum centrifuge and analysed by NanoLC-MS/MS. The samples were then resuspended in 20 μl buffer A (0.1% formic acid) and 1 µl was loaded onto an analytical 25-cm reversed-phase column (Acclaim Pepmap 100 C_18_) with a 75-mm inner diameter (Thermo Fisher Scientific) and separated on the Ultimate 3000 RSLC system coupled via a nano-electrospray source to a Q Exactive HF-X mass spectrometer (Thermo Fisher Scientific). Peptides were separated using a 6–40% gradient of buffer B (80% acetonitrile in 0.1% formic acid) over 123 min at a flow rate of 300 nl/min. Using data-dependent acquisition mode, full MS/MS scans (375–1500 m/z) were performed in the Orbitrap mass analyser (Thermo Fisher Scientific) with a 60,000 resolution at 200 m/z. For the full scans, 3 × 106 ions accumulated within a maximum injection time of 60 ms. The 12 most intense ions with charge states ≥ 2 were sequentially isolated to a target value of 1 × 105 with a maximum injection time of 45 ms and were fragmented by higher-energy collisional dissociation in the collision cell (normalized collision energy 28%) and detected in the Orbitrap mass analyser at a resolution of 30,000. PEAKS Studio v8.5 (Bioinformatics Solutions, Waterloo, ON, Canada) was used to match MS/MS spectra from *X. violacea* and *A. mellifera* venom samples against an in-house database resulting from the annotated transcriptome of each species. Carbamidomethylation was set as a fixed modification, and oxidation of methionine as a variable modification, with a maximum of three missed cleavages for trypsin digestion. Parent and fragment mass error tolerances were set at 5 ppm and 0.015 Da, respectively. A false discovery rate (FDR) of 1% and a unique peptide number ≥ 2 were used to filter out inaccurate proteins. A − 10lgP value > 120 was used to estimate whether detected proteins were identified by a sufficient number of reliable peptides. In order to identify more relevant sequences, the Spider algorithm (PEAKS Studio) was used to find additional mutations or to correct sequences. This algorithm corrects the sequences stored in transcriptomic databases with de novo sequences based on MS/MS spectra, allowing the detection of post-translational modifications (PTMs) and mutations. The minimum ion intensity for PTMs and mutations was set to 5%, and the ALC score was set to ≥ 90 for de novo sequences, leading to low precursor mass errors. Transcripts supported by proteomic data were manually filtered by excluding non-venom-related proteins and peptides, such as house-keeping and structural genes (Additional files 1, 2 and 3). All proteome raw data are accessible via PRIDE (PXD029934, PXD029823, PXD026642) [61–63].

### Genome sequencing

The genomes and annotations of the stingless bees *Tetragobula carbonaria* and *Melipona beecheii* will be published as part of another study, but are already accessible in Genbank (JAUCRC000000000 and JAUCMO000000000) [64]. To sequence the genome of *X. violacea* high molecular weight DNA was extracted from four legs of *X. violacea* adapting the protocol from Miller et al. [65]. Final DNA purity and concentrations were measured using NanoPhotometer® (Implen GmbH, Munich, Germany) and Qubit Fluorometer (Thermo Fisher Scientific, Waltham, MA). Two SMRTbell libraries were constructed following the instructions of the SMRTbell Express Prep kit v2.0 with Low DNA Input Protocol (Pacific Biosciences, Menlo Park, CA). The total input DNA for each library was 1.6 µg. The libraries were loaded at an on-plate concentration of 80 pM using diffusion loading. Two SMRT cell sequencing runs were performed on the Sequel System IIe in CCS mode using 30-h movie time with 2 h pre-extension and sequencing chemistry v2.0. The PacBio sequencing was outsourced to the Genome technology Center Nijmegen, Netherlands. All reads were assembled using HIFIASM assembler [66] after fastq read files of *Xylocopa sp.* were generated by consensus calling of Pacbio HIFI sequencing data using CCS tool (https://github.com/PacificBiosciences/ccs). Reads, which did not take part in the formation of circular consensus sequences, were separated out using in-house developed Perl script (Additional file 36) and were used for closing the gaps with the help of Dentist software [67]. The gap-closed assembly was further polished using Bowtie2 [68], Deepvariant [69], Samtools and BCFtools [70]. Contamination was accounted for by using NCBI Blast and Blobtools [71], and only scaffolds with Arthropoda and No-Hit category were kept. The final gap-closed and contamination free genome of *Xylocopa species* consisted of 353,045,797 bases spread over 3524 scaffolds. The genome was predicted to be 99.7% complete according to the Arthropoda BUSCO gene space (For details see Additional file 10). The genome has been published in GeneBank (SRR21101279) [72].

### Previously published genomes used

The following genomes were used: *Apis cerana* (GCA001442555.1) [73, 74], *Apis dorsata* (GCA000469605.1) [75], *Apis florea* (GCA000184785.2) [76], *Apis mellifera* (GCA003254395.2) [77, 78], *Athalia rosae* (GCA000344095.2) [79], *Bombus terrestris* (GCA000214255.1) [80, 81], *Bombus vosnesenskii* (GCA011952255.1) [82, 83], *Camponotus floridanus* (GCA003227725.1) [84, 85], *Cephus cinctus* (GCA000341935.1) [86], *Ceratina calcarata* (GCA001652005.1) [87], *Colletes gigas* (GCA013123115.1) [88, 89], *Dufourea novaeangliae* (GCA001272555.1) [90], *Eufriesea mexicana* (GCA001483705.1) [91], *Euglossa dilemma* (GCA002201625.1) [92, 93], *Habropoda laboriosa* (GCA001263275.1) [94], *Harpegnathos saltator* (GCA003227715.1) [84, 95], *Linepithema humile* (GCA000217595.1) [96, 97], *Megachile rotundata* (GCA000220905.1) [98], *Megalopta genalis* (GCA011865705.1) [99, 100], *Melipona quadrifasciata* (GCA001276565.1) [101], *Nasonia vitripennis* (GCA009193385.2) [102, 103], *Nomia melanderi* (GCA003710045.1) [104, 105], *Odontomachus brunneus* (GCA010583005.1) [106], *Ooceraea biroi* (GCA003672135.1) [107, 108], *Osmia bicornis* (GCA004153925.1) [109, 110], *Osmia lignaria* (GCA012274295.1) [111], *Polistes canadensis* (GCA_001313835.1) [112, 113], *Polistes dominula* (GCA001465965.1) [114], *Solenopsis invicta* (GCA_016802725.1) [115], *Vollenhovia emeryi* (GCA_000949405.1) [116], and *Wasmannia auropunctata* (GCA000956235.1) [117].

### Genome annotation

We annotated protein-coding genes based on the genome sequence assembly of *C. gigas* (GCA013123115.1, ASM1312311v1). Repeats were soft-masked using RepeatMasker annotations (GCA013123115.1_ASM1312311v1_rm.out) with tabtk, bioawk and seqtk (https://github.com/lh3). We used Funannotate v1.8.1 [118] and Uniprot (sprot) for homology-based evidence based on protein sequences from 11 related bee species: *B. impatiens*: GCF000188095.2, *B. terrestris*: GCF000214255.1, *A. mellifera*: GCF003254395.2, *M. quadrifasciata*: GCA001276565.1, *E. mexicana*: GCF001483705.1, *F. varia* GCA011392965.1, *M. rotundata* GCF000220905.1, *H. laboriosa* GCF001263275.1, *D. novaeangliae* GCF001272555.1, *M. genalis* GCF011865705.1, *N. melanderi* GCF003710045.1. Briefly, funannotate used gene predictions from Genemark-ES, Snap v2006-07–28, glimmerHmm v3.0.4, Augustus v.3.3.3 and CodingQuarry v2.0 together with protein alignments in Evidence Modeler v.1.1.1. Too short, gap-spanning or repeat-overlapping gene models were removed (*n* = 5446) and tRNA genes were detected (*n* = 168) with tRNAscan-SE v2.0.6. Genes were functionally annotated using PFAM v33.1, the UniProt database v2018_11, EggNog (eggnog_4.5/hmmdb databases: Arthropoda, Insecta, Hymenoptera, Drosophila), MEROPS v12.0, CAZYmes in dbCAN v7.0, BUSCO Hymenoptera models v3.0.2, Hymenoptera odb9, SignalP v4.1, and InterProScan5 v81.0. The final annotation contained models for 20,016 protein-coding genes and 168 tRNAs and was estimated to be 87.1% complete (BUSCO4 v4.1.4). The resulting gene annotation files for *C. gigas*, *E. dilemma*, *M. beecheii*, *T. carbonaria* and *Xylocopa violacea* are made available as Additional files 37, 38, 39, 40 and 41 in the Zenodo archive accompanying this manuscript (10.5281/zenodo.7934577).

### Genomic microsynteny analysis

We traced abundant venom gland transcripts that potentially encoded toxins to homologues in the annotated, highly continuous publicly available genomes of bees (and wasps, ants, parasitoid wasps and sawflies as outgroup species) using the online BLAST suite against genomic databases. To identify conserved synteny blocks, we first identified the reciprocal best-match paralogs from hymenopteran all-against-all BLASTP comparisons of the venom genes. Based on the matching sequences, we then extracted exons from the candidate venom genes and their flanking genes. We used those to create local BLAST databases to survey the selected genomes using local tblastx with an e-value cutoff of 0.01. We then applied filters to select venom genes containing scaffolds at least 20 kbp in length (to exclude partial genes) with at least two exons. Where gene annotations were insufficient, we manually re-annotated venom genes where possible, following intron boundaries and using known sequences as templates. This approach allowed us to capture all members of the larger protein families that venom genes belong to——and provided us with outgroup sequences (e.g. trypsins for serine proteases) some of which we used in the tree construction. For flanking genes, we tried to capture up to 5 genes within 100 kbp upstream and downstream from the target to establish reliable synteny. This approach is extremely laborious: while it allowed us to identify every exon homologous to our target gene, we had to manually examine thousands of genomic scaffolds and tens of thousands of genes to make sure we included all the relevant genes into our analysis. We extracted the coding sequences of all complete genes for phylogenetic analysis to establish ortholog groups in addition to their microsyntenic patterns. All resulting annotations are available as part of the Additional Materials (Additional file 42). It is important to note that we could not correct for the assembly quality of the genomes used.

### Orthology prediction and phylogenetic analysis

All toxin transcripts together with toxin genes and their outgroup venom-unrelated homologues (e.g. trypsins and chymotrypsins in case of serine proteases) were arranged by gene family and aligned as translated amino acids using MAFFT [119] (L-INS-I, 1000 iterations). Name convention was established to differentiate between genomic sequences (first two letters of both genus and species name, followed by the last three digits of a bioinformatic scaffold ID, followed—if applicable—by an abbreviation of a pre-existing gene annotation, followed by letters a to z to differentiate between sequences from the same scaffold); proteo-transcriptomic sequences (names kept the same as generated by transcriptome assemblers); homologues from UniProt and SwissProt databases used to provide outgroups and fill the gaps in sequence space (kept as UniProt or SwissProt IDs, but reduced to 10 characters if needed due to strict limitations of phylip format used by Exabayes). Alignments were manually inspected for overt errors (e.g. proper alignment of the cysteine backbone) and used to construct phylogenetic trees in Exabayes [120] (four parallel runs of four chains each, runs stopped when average standard deviation of split frequencies of trees reached below 5%). Resulting trees are shown in the Additional files 12, 13, 14,  15, 20, 22, 24, 26, 28 and 31, with toxin sequences recovered from *Apis, Halictus* or *Xylocopa* venom marked as red arrows and non-toxic physiological sequences marked with grey arrow.

### A novel perspective on relations of short peptides: embedding space analysis

Every year, algorithms improve natural language processing (NLP) tasks such as automated translation or question answering, in particular by feeding large text corpora into Deep Learning (DL)-based Language Models (LMs) [121]. These advances have been transferred to protein sequences by learning to predict masked or missing amino acids using large databases of raw protein sequences as input [122, 123]. Such methods leverage the wealth of information present in exponentially growing unlabelled protein sequence databases by solely relying on sequential patterns found in the input. Processing the information learned by such protein LMs (pLMs), e.g., by feeding a protein sequence as input to the network and constructing vectors thereof from the activation in the network’s last layers, yields a representation of protein sequences referred to as embeddings [122]. This way, features learned by the pLM can be transferred to any (prediction) task requiring numerical protein representations (transfer learning) which has already been showcased for various aspects ranging from protein structure [124] over protein function [125]. Further, it was shown that distance in embedding space correlates with protein function and can be used as an orthogonal signal for clustering proteins into functional families [125].

Here, we used the pLM ProtT5-XL-UniRef50 [122] (in the following ProtT5) to create fixed-length vector representations for each protein sequence (per-protein embeddings) irrespective of its length. Towards this, we first created individual vector representations for each residue in a protein. In order to derive fixed-length vector representations for single proteins (per-protein embedding) irrespective of a protein’s length, we then averaged over all residue embeddings in a protein (Fig. 1 in Elnaggar et al. [122]). The protein Language Model (pLM) ProtT5 was trained solely on unlabelled protein sequences from BFD (Big Fantastic Database; 2.5 billion sequences including metagenomic sequences) [126] and UniRef50. ProtT5 has been built in analogy to the NLP (Natural Language Processing) T5 [121] ultimately learning some of the constraints of protein sequence. As ProtT5 was only trained on unlabelled protein sequences and no supervised training or fine-tuning was performed, there is no risk of information leakage or overfitting to a certain class or label. As a result, every protein was represented as 1024-dimensional per-protein embeddings. Those high-dimensional representations were projected to 3 days using UMAP (n_neighbors = 25, min_dist = 0.5, random_state = 42, n_components = 3) or PCA and coloured according to their respective group to allow for visual analysis. Embeddings and 3-day plots were created using the bio_embeddings package [127]. All information on sequences used in the machine learning approach is specified in the additional material in a general table (Additional file 33). Unaligned mature and full sequences are given as fasta files (Additional files 43 and 44). However, the sequence data set for the ML approach differs from the sequence set used for phylogenetic analyses in that respect, that only unique mature sequences can be used. Sequence similarities for alignments (Additional file 45) were calculated by dividing the aligned sequence length by the number of sequences that align with a BLOSUM62 score of above 0, multiplied by 100. Global alignments were reconstructed with the Needleman–Wunsch algorithm (script attached, BLOSUM62 matrix, gap_penality = -10, gap_extension_penality = -0.5). Subfamily sequence similarity was calculated by taking the average over all pairwise sequence similarities between all possible pairs within the groups with standard deviation. The trees of mature sequences for ML analysis (Additional file 46) were reconstructed using the settings from above. Interactive 3D plots of protein spaces are given in Additional file 11 and were reconstructed using the algorithm deposited on github: https://github.com/Rostlab/RostSpace

### Supplementary Information

**Additional file 1.** Proteo-transcriptomically identified venom components in *X. violacea*.

**Additional file 2.** Proteo-transcriptomically identified venom components in *H. scabiosae*.

**Additional file 3.** Proteo-transcriptomically identified venom components in *X. violacea*.

**Additional file 4.** VG Assembly file of *Xylocopa violacea* following ORF prediction by Transdecoder.

**Additional file 5.** VG Assembly file of *Halictus scabiosae* following ORF prediction by Transdecoder.

**Additional file 6.** VG Assembly file of *Apis mellifera* following ORF prediction by Transdecoder.

**Additional file 7.** BUSCO statistics for venom gland assemblies of *X. violacea, H. scabiosae*, *A. mellifera.*

**Additional file 8.** Listed bioactivity and description of the prevalent bee venom proteins.

**Additional file 9.** List of mined and used high-quality hymenopteran genomes.

**Additional file 10.** Resulting statistics of the new genome sequence of *X. violacea*.

**Additional file 11.** Interactive 3D protein spaces that Figs. 3 and 7 are based upon.

**Additional file 12.** Phylogenetic tree of phospholipase A2 proteins, rerooted on an outgroup. Red arrows mark those that were recovered from the transcriptomes of *X. violacea*, *H. scabiosae* and *A. mellifera* in the present study. Genomic sequences recovered in the present study have naming convention of Gesp###a where Ge stands for first two letters of the genus name, sp stands for first two letters of the species name, ### stands for the last three digits of the genomic scaffold ID, and a stands for A to Z identifier given to homologous genes if several were found on the same continuous genomic scaffold. Where genomic and transcriptomic sequences were identical, we kept transcriptomic sequence. UniProt and GeneBank IDs were kept in their original form.

**Additional file 13.** Phylogenetic tree of the Hyaluronidase protein family, rerooted on an outgroup. Red arrows mark those that were recovered from the transcriptomes of *X. violacea, H. scabiosae* and *A. mellifera* in the present study. Genomic sequences recovered in the present study have naming convention of Gesp### where Ge stands for first two letters of the genus name, sp stands for first two letters of the species name, ### stands for the last three digits of the genomic scaffold ID. Where genomic and transcriptomic sequences were identical, we kept transcriptomic sequence. UniProt and Gene- Bank IDs were kept in their original form.

**Additional file 14.** Phylogenetic tree of the Icarapin protein family, rerooted according to an outgroup. Red arrows mark those that were recovered from the transcriptomes of *X. violacea*, *H. scabiosae* and *A. mellifera* in the present study. Genomic sequences recovered in the present study have naming convention of Gesp### where Ge stands for first two letters of the genus name, sp stands for first two letters of the species name, ### stands for the last three digits of the genomic scaffold ID. Where genomic and transcriptomic sequences were identical, we kept transcriptomic sequence.

**Additional file 15.** Phylogenetic tree of the Dipeptidyl peptidase-4 protein family. Red arrows mark those that were recovered from the transcriptomes of *X. violacea*, *H. scabiosae* and *A. mellifera* in the present study. Genomic sequences recovered in the present study have naming convention of Gesp###PPPP## where Ge stands for first two letters of the genus name, sp stands for first two letters of the species name, ### stands for the last three digits of the genomic scaffold ID, PPPP## stands for the protein label. Where genomic and transcriptomic sequences were identical, we kept transcriptomic sequence. UniProt and GeneBank IDs were kept in their original form.

**Additional file 16.** Alignment of phospholipase A2 proteins.

**Additional file 17.** Alignment of hyaluronidase proteins.

**Additional file 18.** Alignment of icarapin proteins.

**Additional file 19.** Alignment of dipeptidyl peptidase 4 proteins.

**Additional file 20.** Phylogenetic tree of the Acid Phosphatase protein family, rerooted according to an outgroup. Red arrows mark those that were recovered from the transcriptomes of *X. violacea*, *H. scabiosae* and *A. mellifera* in the present study. Genomic sequences recovered in the present study have naming convention of Gesp###PPa(U) where Ge stands for first two letters of the genus name, sp stands for first two letters of the species name, ### stands for the last three digits of the genomic scaffold ID, PP stands for the protein label and a stands for A to Z identifier given to homologous genes if several were found on the same continuous genomic scaffold. Capital U at the end of the name indicates that gene homology was not proposed prior to this study. Where genomic and transcriptomic sequences were identical, we kept transcriptomic sequence. UniProt and GeneBank IDs were kept in their original form.

**Additional file 21.** Alignment of venom acid phosphatase proteins.

**Additional file 22.** Phylogenetic tree of the Serine Protease protein family, rerooted according to an outgroup. Red arrows mark those that were recovered from the transcriptomes of *X. violacea*, *H. scabiosae* and *A. mellifera* in the present study. Genomic sequences recovered in the present study have naming convention of Gesp###PPa(U) where Ge stands for first two letters of the genus name, sp stands for first two letters of the species name, ### stands for the last three digits of the genomic scaffold ID, PP stands for the protein label and a stands for A to Z identifier given to homologous genes if several were found on the same continuous genomic scaffold. Capital U at the end of the name indicates that gene homology was not proposed prior to this study. Where genomic and transcriptomic sequences were identical, we kept transcriptomic sequence. UniProt and GeneBank IDs were kept in their original form.

**Additional file 23.** Alignment of venom serine protease proteins.

**Additional file 24.** Phylogenetic tree of the Venom Allergens protein family, rerooted according to an outgroup. Red arrows mark those that were recovered from the transcriptomes of *X. violacea*, *H. scabiosae* and *A. mellifera* in the present study. Genomic sequences recovered in the present study have naming convention of Gesp###PPa(U) where Ge stands for first two letters of the genus name, sp stands for first two letters of the species name, ### stands for the last three digits of the genomic scaffold ID, PP stands for the protein label and a stands for A to Z identifier given to homologous genes if several were found on the same continuous genomic scaffold. Capital U at the end of the name indicates that gene homology was not proposed prior to this study. Where genomic and transcriptomic sequences were identical, we kept transcriptomic sequence. UniProt and GeneBank IDs were kept in their original form.

**Additional file 25.** Alignment of venom allergen proteins.

**Additional file 26.** Phylogenetic tree of the Secapin protein family, rerooted according to an outgroup. Red arrows mark those that were recovered from the transcriptomes of *X. violacea*, *H. scabiosae* and *A. mellifera* in the present study. Genomic sequences recovered in the present study have naming convention of Gesp###PPPP where Ge stands for first two letters of the genus name, sp stands for first two letters of the species name, ### stands for the last three digits of the genomic scaffold ID, PPPP stands for the protein label. Where genomic and transcriptomic sequences were identical, we kept transcriptomic sequence. UniProt and GeneBank IDs were kept in their original form.

**Additional file 27.** Alignment of secapin proteins.

**Additional file 28.** Phylogenetic tree of anthophilin1 peptides. Phylogenetic tree of Anthophilin1 protein family. Red arrows mark those that were recovered from the transcriptomes of *X. violacea*, *H. scabiosae* and *A. mellifera* in the present study. Genomic sequences recovered in the present study have naming convention of Gesp###_NAME_# where Ge stands for first two letters of the genus name, sp stands for first two letters of the species name, ### stands for the last three digits of the genomic scaffold ID, NAME stands for the protein label and # stands for numerical identifier given to homologous genes if several were found on the same continuous genomic scaffold. Where genomic and transcriptomic sequences were identical, we kept transcriptomic sequence.

**Additional file 29.** Alignment of anthophilin1 peptides.

**Additional file 30.** Alignment of *Apis* melittin sequence with *Vollenhovia*.

**Additional file 31.** Phylogenetic tree of melittin peptides. Phylogenetic tree of the Melittin protein family. Red arrows mark those that were recovered from the transcriptomes of *X. violacea*, *H. scabiosae* and *A. mellifera* in the present study. Genomic sequences recovered in the present study have naming convention of Gesp###PPa(U) where Ge stands for first two letters of the genus name, sp stands for first two letters of the species name, ### stands for the last three digits of the genomic scaffold ID, PP stands for the protein label and a stands for A to Z identifier given to homologous genes if several were found on the same continuous genomic scaffold. Where genomic and transcriptomic sequences were identical, we kept transcriptomic sequence. UniProt and GeneBank IDs were kept in their original form. Forms known from proteomes only were kept with the names given in the original studies.

**Additional file 32.** Alignment of melittin peptides.

**Additional file 33.** Information on all sequences used for the ML approach.

**Additional file 34.** Dataset of “aculeatoxins” with signal peptide from Robinson et al.

**Additional file 35.** Dataset of only mature regions of “aculeatoxins” from Robinson et al.

**Additional file 36.** Custom Pearl script used to separate the reads.

**Additional file 37.** Genome annotation of *Colletes gigas*.

**Additional file 38**. Genome annotation of *Euglossa dilemma*.

**Additional file 39.** Genome annotation of *Tetragonula carbonaria*.

**Additional file 40.** Genome annotation of *Melipona beecheii*.

**Additional file 41.** Genome annotation of Xylocopa violacea.

**Additional file 42.** Gff files of all toxin gene annotations.

**Additional file 43.** Unaligned sequences for the ML approach (full sequences).

**Additional file 44.** Unaligned sequences for the ML approach (mature sequences).

**Additional file 45.** Similarity Matrices for all venom proteins (mature sequences) in ML approach.

**Additional file 46.** All phylogenetic trees for all venom proteins (mature sequences) in ML approach.

## Data Availability

All data generated or analysed during this study are included in this published article, its supplementary information files and publicly available repositories. All proteome data are available in PRIDE via ProtXChange (PXD029934, PXD029823, PXD026642). In NCBI all transcriptome data (SRR14690757, SRR14690758, SRR14690759) and genome data (SRR21101279, JAUCRC000000000, JAUCMO000000000) are made accessible via the SRA archive. All other data that is not obligatory to submission (e.g. assemblies and genome annotations) are provided open access alongside the supplementary data as additional data files in the database Zenodo under the https://doi.org/10.5281/zenodo.8052397.

## Abbreviations

APH: Acid phosphatase
DL: Deep learning
DPP4: Dipepditylpeptidase 4
LM: Language model
MCDP: Mast cell degranulating protein
MOXD1: Monooxygenase DBH-like1 gene
MS: Mass spectrometry
NLP: Natural language processing
ORF: Open reading frame
PBVP: Prevalent bee venom proteins
pLM: Protein langauage model
Pr: Prolin rich
PTM: Post-translational modification
TBC11: TBC1 domain family member gene
TPM: Transcript per million
VSP: Venom serine proteases
