# Supplementary material for: Prevalent bee venom genes evolved before the aculeate stinger and eusociality
Source: BMC Biol. 2023 Oct 23;21:229. doi: 10.1186/s12915-023-01656-5 (PMC10591384; doi:10.1186/s12915-023-01656-5)
Supplement: Supplementary file 2 — Additional file 2. Proteo-transcriptomically identified venom components in H. scabiosae. [file 12915_2023_1656_MOESM2_ESM.pdf]

Halictus scabiosae

Transcriptomic data analysis

Transdecoder protein IDs	Transcript ID	Expression value	Blast-P toxprot (sseqid)	E-value	Bitscore	Blast-P UniProt Apocrita (sseqid)	E-value	Bitscore	Blast-P UniProt All (sseqid)	E-value	Bitscore	Physiological association	Venom protein family
J646877.p1	J646877	29.27139316	NA	NA	NA	NA	NA	NA	Protein translation factor SUI1 like protein	3.33E-78	230	No venom function	NA
J647002.p1	J647002	0.168754513	NA	NA	NA	NA	NA	NA	Peptidylprolyl isomerase	2.91E-141	400	No venom function	NA
NODE_11040_length_424_cov_3.833811_g10395_i0.p1	NODE_11040_length_424_cov_3.833811_g10395_i0	1.121949383	NA	NA	NA	NA	NA	NA	Apolipophorins	2.32E-47	169	No venom function	NA
NODE_11359_length_430_cov_8.613333_g10819_i0.p1	NODE_11359_length_430_cov_8.613333_g10819_i0	2.0324027	NA	NA	NA	NA	NA	NA	Apolipophorin (Fragment)	2.09E-56	195	No venom function	NA
NODE_1159_length_1441_cov_15.445095_g901_i0.p1	NODE_1159_length_1441_cov_15.445095_g901_i0	3.067365532	NA	NA	NA	NA	NA	NA	Transgelin	2.64E-130	370	No venom function	NA
NODE_14217_length_383_cov_79.085366_g13669_i0.p2	NODE_14217_length_383_cov_79.085366_g13669_i0	27.77990047	Venom serine protease inhibitor	8.71E-12	52.4	Chymotrypsin inhibitor	2.29E-20	70.9	Venom serine protease inhibitor	8.95E-26	95.9	Venom component	Venom serine protease inhibitor
NODE_145_length_3358_cov_30.248553_g77_i1.p1	NODE_145_length_3358_cov_30.248553_g77_i1	4.786309015	NA	NA	NA	NA	NA	NA	Dolichyl-diphosphooligosaccharide--protein	0	1411	No venom function	NA
NODE_150_length_3308_cov_392.762140_g82_i0.p1	NODE_150_length_3308_cov_392.762140_g82_i0	70.10773263	NA	NA	NA	Elongation factor 1-alpha	0.00000265	43.5	Elongation factor 1-alpha	0	1732	Venom component?	EF1 alpha
NODE_15838_length_356_cov_2.583630_g15179_i0.p1	NODE_15838_length_356_cov_2.583630_g15179_i0	1.242733601	L-amino-acid oxidase	4.53E-12	56.2	NA	NA	NA	Smox_0 protein	1.80E-24	100	No venom function	Venom component
NODE_1794_length_1216_cov_13.130060_g1569_i0.p1	NODE_1794_length_1216_cov_13.130060_g1569_i0	1.717444945	NA	NA	NA	NA	NA	NA	Glyoxalase domain-containing protein 4	0	506	No venom function	NA
NODE_18897_length_323_cov_3.048387_g18238_i0.p1	NODE_18897_length_323_cov_3.048387_g18238_i0	2.432877846	NA	NA	NA	NA	NA	NA	Thioredoxin-dependent peroxide mitochond	4.34E-66	204	No venom function	NA
NODE_1926_length_1102_cov_536.289192_g1566_i0.p1	NODE_1926_length_1102_cov_536.289192_g1566_i0	98.65430645	Translationally-controlled tumor protein ho	6.81E-57	173	NA	NA	NA	Translationally-controlled tumor protein lik	2.48E-115	329	No venom function	NA
NODE_1966_length_1151_cov_15.059307_g1725_i0.p1	NODE_1966_length_1151_cov_15.059307_g1725_i0	1.154304537	NA	NA	NA	Major royal jelly protein 1	4.37E-91	273	Major royal jelly protein 2	2.38E-113	341	Venom component	Major royal jelly
NODE_19706_length_317_cov_5.557252_g19153_i0.p1	NODE_19706_length_317_cov_5.557252_g19153_i0	1.863806317	NA	NA	NA	NA	NA	NA	Putative polyamine oxidase 5 (Fragment)	3.43E-37	135	No venom function	NA
NODE_19842_length_316_cov_3.639847_g19289_i0.p1	NODE_19842_length_316_cov_3.639847_g19289_i0	1.88595667	NA	NA	NA	NA	NA	NA	Apolipophorin (Fragment)	1.81E-30	119	No venom function	NA
NODE_2013_length_1139_cov_12.987085_g1769_i0.p1	NODE_2013_length_1139_cov_12.987085_g1769_i0	2.257870907	Phospholipase A2	1.51E-40	132	Phospholipase A2	1.60E-41	132	Phospholipase A2	4.83E-75	231	Toxin	Phospholipase A2
NODE_203_length_2843_cov_39.225434_g127_i0.p1	NODE_203_length_2843_cov_39.225434_g127_i0	3.085572152	NA	NA	NA	NA	NA	NA	Cytosol aminopeptidase	0	832	No venom function	NA
NODE_2036_length_1134_cov_232.479147_g1790_i0.p1	NODE_2036_length_1134_cov_232.479147_g1790_i0	20.85148102	NA	NA	NA	NA	NA	NA	Guanine nucleotide-binding protein subunit	0	645	No venom function	NA
NODE_2049_length_1130_cov_16.738605_g1802_i0.p1	NODE_2049_length_1130_cov_16.738605_g1802_i0	2.823002907	NA	NA	NA	NA	NA	NA	Aldose reductase	0	605	No venom function	NA
[illegible]	[illegible]	[illegible]	[illegible]	[illegible]	[illegible]	[illegible]	[illegible]	[illegible]	[illegible]	[illegible]	[illegible]	[illegible]	[illegible]

Proteome data analysis

Checked	Protein FDR C	Master	Accession	Description	Exp. q-value:	Coverage [%]	# Peptides	# PSMs	# Unique Pep	# AAs	MW [kDa]	calc. pI	Score Mascot	# Peptides (by Search Engine): Mascot
FALSE	High	Master Protein	J646877.p1 GENE.J646877~~J	PF01253.22 2e-27 J646877:688-102	0.003	35	3	4	3	110	12.4	6.77	52	3
FALSE	High	Master Protein	J647002.p1 GENE.J647002~~J	PF00254.28 3.6e-36,EF-hand_1|PF0	0	44	9	15	9	239	26.5	4.79	290	9
FALSE	High	Master Protein	NODE_11040_length_424_co	PF01347.22 1.8e-12,DUF2202|PF09	0	31	4	12	1	140	14.8	8.9	280	4
FALSE	High	Master Protein	NODE_11359_length_430_co	PF04357.13 5,TamB|PF04357.13|1.	0	30	4	7	4	143	15.6	9.09	154	4
FALSE	High	Master Protein	NODE_1159_length_1441_co	PF00307.31 3.5e-23,CH|PF00307.31	0	33	5	7	5	236	26.4	9.07	127	5
FALSE	High	Master Protein	NODE_14217_length_383_co	PF01826.17 5.1e-13,cEGF|PF12662.	0	34	2	8	2	85	8.8	4.82	254	2
FALSE	High	Master Protein	NODE_145_length_3358_cov	PF02516.14 8.8e-141,STT3|PF0251	0.004	4	3	3	3	705	80.1	8.13	56	3
FALSE	High	Master Protein	NODE_150_length_3308_cov	PF00009.27 2e-66,EFG_IV|PF03764	0	15	10	16	10	844	94.6	6.57	150	10
FALSE	High	Master Protein	NODE_15838_length_356_co	PF01593.24 5.2e-17,NAD_binding_8	0	37	2	4	2	90	9.6	4.78	137	2
FALSE	High	Master Protein	NODE_1794_length_1216_co	PF00903.25 0.0016,Glyoxalase|PF0	0	15	4	7	4	281	32	5.76	71	4
FALSE	High	Master Protein	NODE_18897_length_323_co	PF00578.21 6.9e-33,Redoxin|PF085	0.004	39	3	3	3	107	12	7.33	57	3
FALSE	High	Master Protein	NODE_1926_length_1102_co	PF00838.17 1.2e-62,PfUIS3|PF1156	0	31	4	6	4	172	19.8	4.81	104	4
FALSE	High	Master Protein	NODE_1966_length_1151_co	PF03022.16 5.9e-64,NHL|PF01436.2	0	34	9	16	9	353	38.9	5.41	222	9
FALSE	High	Master Protein	NODE_19706_length_317_co	PF01593.24 4.1e-17,Phage_tail_2|P	0.004	51	4	6	4	92	10.2	6.79	62	4
FALSE	High	Master Protein	NODE_19842_length_316_co	GENE.NODE_19842_length_316_cov_3	0	38	4	7	4	105	11.6	8.97	183	4
FALSE	High	Master Protein	NODE_2013_length_1139_co	PF05826.12 2.8e-37,Phospholip_A2_	0	33	6	8	6	216	24.8	5.83	88	6
FALSE	High	Master Protein	NODE_203_length_2843_cov	PF00883.21 2.7e+03,Peptidase_M1	0	35	13	25	13	509	54.7	7.12	241	13
FALSE	High	Master Protein	NODE_2036_length_1134_co	PF00400.32 0.00041,WD40|PF0040	0.004	30	7	8	7	317	35.6	7.74	61	7
FALSE	High	Master Protein	NODE_2049_length_1130_co	PF00248.21 9.1e-54 NODE_2049_le	0	34	9	13	9	317	35.7	7.46	110	9
[illegible]	[illegible]	[illegible]	[illegible]	[illegible]	[illegible]	[illegible]	[illegible]	[illegible]	[illegible]	[illegible]	[illegible]	[illegible]	[illegible]	[illegible]
