# Supplementary material for: Prevalent bee venom genes evolved before the aculeate stinger and eusociality
Source: BMC Biol. 2023 Oct 23;21:229. doi: 10.1186/s12915-023-01656-5 (PMC10591384; doi:10.1186/s12915-023-01656-5)
Supplement: Supplementary file 8 — Additional file 8. Listed bioactivity and description of the prevalent bee venom proteins. [file 12915_2023_1656_MOESM8_ESM.pdf]

Venom component	Function	Activity	Reference
Apamin	Toxin	Neurotoxic	Apamin blocks certain neurotransmitter-induced increases in potassium permeability. B. E. C. Banks, C. Brown, G. M. Burgess, G. Burnstock, M. Claret, T. M. Cocks & D. H. Jenkinson. Nature volume 282, pages 415–417 (1979)<br>Apamin, a neurotoxin specific for one class of Ca2+-dependent K+ channels. M Lazdunski.Cell Calcium. 1983. Dec;4(5-6):421-8.
Mast cell degranulating protein	Venom component	Neurotoxic, Histamin release	Handbook of Biologically Active Peptides (Second Edition), Chapter 58 - Hymenoptera Insect Peptides, 2013, Pages 416-422, Mario SergioPalma<br>xPharm: The Comprehensive Pharmacology Reference, 2007, Pages 1-3, Mast Cell Degranulating Peptide, Mark A.Simmons
Melittin	Toxin	Cytolytic, disrupts surface tension, relea	Biosci Rep (2007) 27:189–223 DOI 10.1007/s10540-006-9030-z, REVIEW ARTICLE, Melittin: a Membrane-active Peptide with Diverse Functions, H. Raghuraman Æ Amitabha Chattopadhyay<br>Review, Bee Venom: An Updating Review of Its Bioactive Molecules and Its Health Applications, Maria Carpena , Bernabe Nuñez-Estevez , Anton Soria-Lopez and Jesus Simal-Gandara, Nutrients, MDPI, 2020
Secapin	Venom component	Antifibronilytic	Secapin, a bee venom peptide, exhibits anti-fibrinolytic, anti-elastolytic, and anti-microbial activities. Kwang SikLeea1Bo YeonKima1Hyung JooYoonbYong SooChoibByung RaeJina. Developmental & Comparative Immunology. Volume 63, October 2016, Pages 27-35
Hyaluronidase	Toxin	Spreading factor, pore forming	Marković-Housley Z., Miglierini G., Soldatova L., Rizkallah P.J., Müller U., Schirmer T. Crystal structure of hyaluronidase, a major allergen of bee venom. Structure. 2000;8:1025–1035. doi: 10.1016/S0969-2126(00)00511-6. [PubMed] [CrossRef] [Google Scholar] [Ref list]<br>Diversity of peptidic and proteinaceous toxins from social Hymenoptera venoms. Dos Santos-Pinto JRA, Perez-Riverol A, Lasa AM, Palma MS. Toxicon. 2018 Jun 15; 148():172-196.<br>Bee Venom: An Updating Review of Its Bioactive Molecules and Its Health Applications. Maria Carpena, Bernabe Nuñez-Estevez, Anton Soria-Lopez, and Jesus Simal-Gandara. Nutrients. 2020 Nov; 12(11): 3360. Published online 2020 Oct 31. doi: 10.3390/nu12113360. PMCID: PMC7693387. PMID: 33142794
Icarapin	Venom component	Undefined	NA
Phospholipase A2	Toxin	Neurotoxic,	Localization of structural elements of bee venom phospholipase A2 involved in N-type receptor binding and neurotoxicity. J P Nicolas 1 , Y Lin, G Lambeau, F Ghomashchi, M Lazdunski, M H Gelb. J Biol Chem. 1997 Mar 14;272(11):7173-81. doi: 0.1074/jbc.272.11.7173.<br>Review, Bee Venom: An Updating Review of Its Bioactive Molecules and Its Health Applications, Maria Carpena , Bernabe Nuñez-Estevez , Anton Soria-Lopez and Jesus Simal-Gandara, Nutrients, MDPI, 2020
Venom antigen 3/5	Venom component	Undefined	NA
Venom dipeptidyl peptidase 4	Venom component	Processses venom components e.g. me	Bee Venom: An Updating Review of Its Bioactive Molecules and Its Health Applications. Maria Carpena, Bernabe Nuñez-Estevez, Anton Soria-Lopez, and Jesus Simal-Gandara. Nutrients. 2020 Nov; 12(11): 3360. Published online 2020 Oct 31. doi: 10.3390/nu12113360. PMCID: PMC7693387. PMID: 33142794
Venom acid phosphatase	Venom component	Histamin release	Bee Venom: An Updating Review of Its Bioactive Molecules and Its Health Applications. Maria Carpena, Bernabe Nuñez-Estevez, Anton Soria-Lopez, and Jesus Simal-Gandara. Nutrients. 2020 Nov; 12(11): 3360. Published online 2020 Oct 31. doi: 10.3390/nu12113360. PMCID: PMC7693387. PMID: 33142794
Venom serine protease	Venom component	Lethal Melanization repsonse,	Dual Function of a Bee Venom Serine Protease: Prophenoloxidase-Activating Factor in Arthropods and Fibrin(ogen)olytic Enzyme in Mammals. Young Moo Choo1., Kwang Sik Lee1., Hyung Joo Yoon2, Bo Yeon Kim1, Mi Ri Sohn1, Jong Yul Roh3, Yeon Ho Je3, Nam Jung Kim2, Iksoo Kim4, Soo Dong Woo5, Hung Dae Sohn1, Byung Rae Jin1. PLOS one 2010. 5 (5)
Tertiapin	Toxin	Neurotoxic	*Gauldie, J; Hanson, JM; Rumjanek, FD; Shipolini, RA; Vernon, CA (1976). "The peptide components of bee venom". European Journal of Biochemistry.* ***61*** *(2): 369–376. doi:10.1111/j.1432-1033.1976.tb10030.x. PMID 1248464.*<br>The Effect of Bee Venom Peptides Melittin, Tertiapin, and Apamin on the Human Erythrocytes Ghosts: A Preliminary Study. Świątły-Błaszkiewicz, A.; Mrówczyńska, L.; Matuszewska, E.; Lubawy, J.; Urbański, A.; Kokot, Z.J.; Rosiński, G.; Matysiak, J. *Metabolites* **2020**, *10* , 191. https://doi.org/10.3390/metabo10050191
