# Supplementary material for: Prevalent bee venom genes evolved before the aculeate stinger and eusociality
Source: BMC Biol. 2023 Oct 23;21:229. doi: 10.1186/s12915-023-01656-5 (PMC10591384; doi:10.1186/s12915-023-01656-5)
Supplement: Supplementary file 14 — Additional file 14. Phylogenetic tree of the Icarapin protein family, rerooted according to an outgroup. Red arrows mark those that were recovered from the transcriptomes of X. violacea, H. scabiosae and A. mellifera in the present study. Genomic sequences recovered in the present study have naming convention of Gesp### where Ge stands for first two letters of the genus name, sp stands for first two letters of the species name, ### stands for the last three digits of the genomic scaffold ID. Where genomic and transcriptomic sequences were identical, we kept transcriptomic sequence. [file 12915_2023_1656_MOESM14_ESM.pdf]

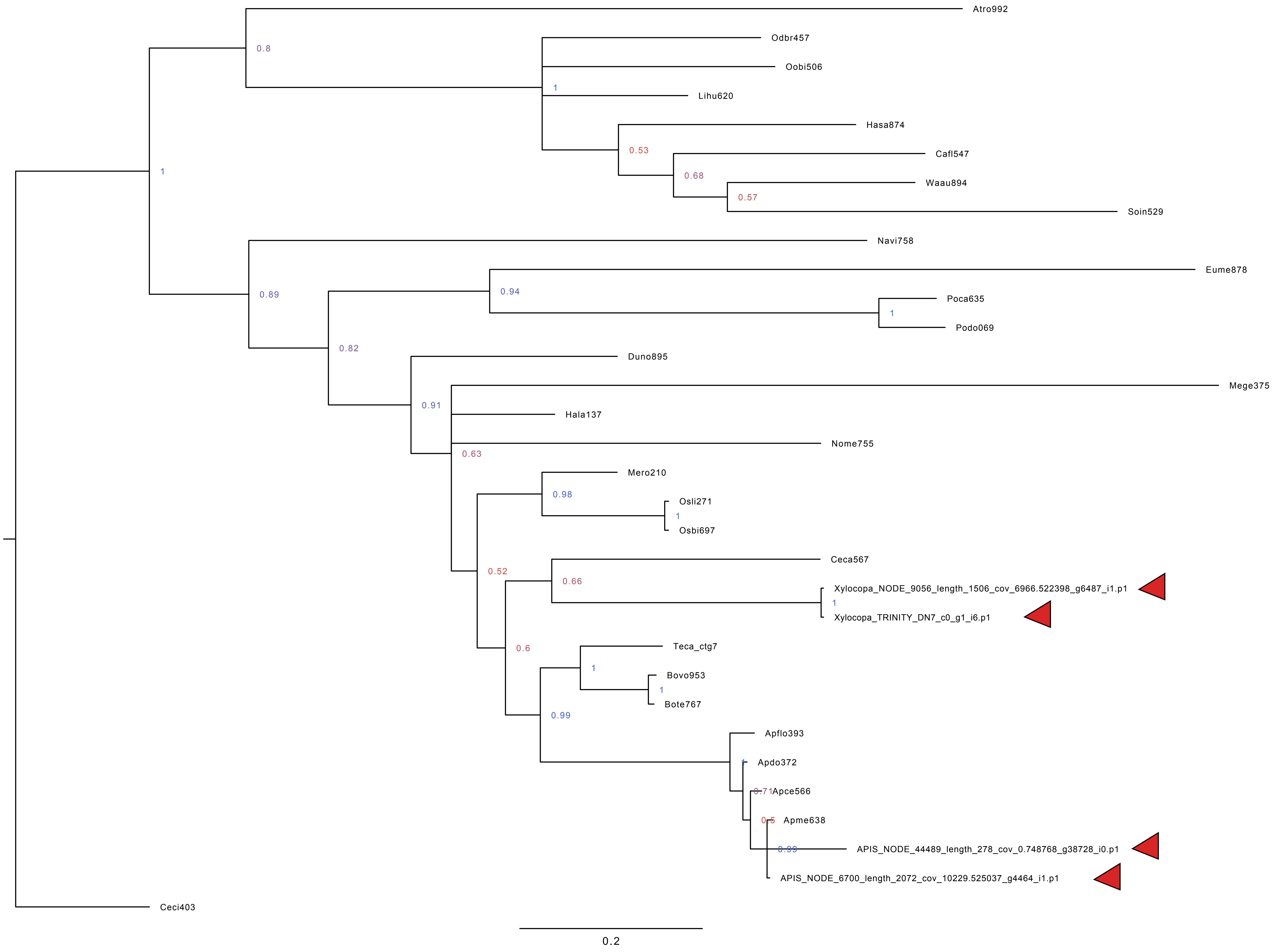


**Phylogenetic tree of the Icarapin protein family, rerooted according to an outgroup.** Red arrows mark those that were recovered from the transcriptomes of *X. violacea*, *H. scabiosae* and *A. mellifera* in the present study. Genomic sequences recovered in the present study have naming convention of *Gesp###* where *Ge* stands for first two letters of the genus name, *sp* stands for first two letters of the species name, *###* stands for the last three digits of the genomic scaffold ID. Where genomic and transcriptomic sequences were identical, we kept transcriptomic sequence.
