# Supplementary material for: Prevalent bee venom genes evolved before the aculeate stinger and eusociality
Source: BMC Biol. 2023 Oct 23;21:229. doi: 10.1186/s12915-023-01656-5 (PMC10591384; doi:10.1186/s12915-023-01656-5)
Supplement: Supplementary file 28 — Additional file 28. Phylogenetic tree of anthophilin1 peptides. Phylogenetic tree of Anthophilin1 protein family. Red arrows mark those that were recovered from the transcriptomes of X. violacea, H. scabiosae and A. mellifera in the present study. Genomic sequences recovered in the present study have naming convention of Gesp###_NAME_# where Ge stands for first two letters of the genus name, sp stands for first two letters of the species name, ### stands for the last three digits of the genomic scaffold ID, NAME stands for the protein label and # stands for numerical identifier given to homologous genes if several were found on the same continuous genomic scaffold. Where genomic and transcriptomic sequences were identical, we kept transcriptomic sequence. [file 12915_2023_1656_MOESM28_ESM.pdf]

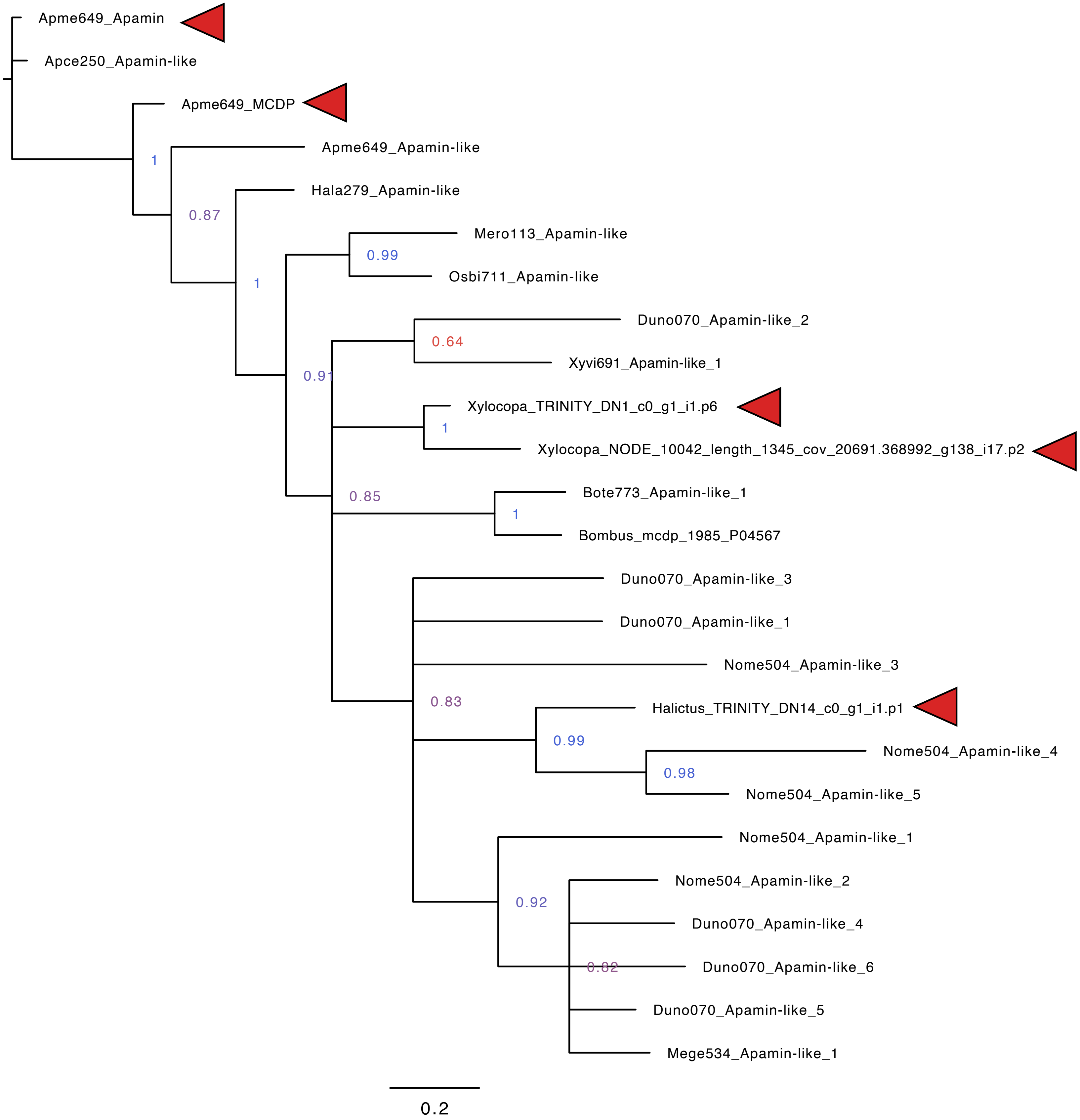


**Phylogenetic tree of Anthophilin1 protein family.** Red arrows mark those that were recovered from the transcriptomes of *X. violacea*, *H. scabiosae* and *A. mellifera* in the present study. Genomic sequences recovered in the present study have naming convention of *Gesp###_NAME_#* where *Ge* stands for first two letters of the genus name, *sp* stands for first two letters of the species name, *###* stands for the last three digits of the genomic scaffold ID, *NAME* stands for the protein label and # stands for numerical identifier given to homologous genes if several were found on the same continuous genomic scaffold. Where genomic and transcriptomic sequences were identical, we kept transcriptomic sequence.
