# Supplementary material for: Prevalent bee venom genes evolved before the aculeate stinger and eusociality
Source: BMC Biol. 2023 Oct 23;21:229. doi: 10.1186/s12915-023-01656-5 (PMC10591384; doi:10.1186/s12915-023-01656-5)
Supplement: Supplementary file 3 — Additional file 3. Proteo-transcriptomically identified venom components in X. violacea. [file 12915_2023_1656_MOESM3_ESM.pdf]

Xylocopa violacea

Transdecoder protein IDs	Transcript ID	Expression value	Blast-P toxprot (sseqid)	E-value	Bitscore	Blast-P UniProt Apocrita (sseqid)	E-value	Bitscore	Blast-P UniProt All (sseqid)	E-value	Bitscore	Physiological association	Venom protein family	Protein Group	Protein ID	Accession	-10lgP	Coverage (%)	#Peptides	#Unique	PTM	Avg. Mass	Description
			Transcriptome data analysis											Proteome data analysis									
J678067.p1	J678067	4.49562464	NA	NA	NA	NA	NA	NA	Uncharacterized protein/putative sodium-co	4.47E-154	452	No venom function	NA	94	67	J678067.p1	122.12	14	4	4		51609	GENE.J678067~~J678067.p1 ORF type:complete len:514 (+) score=119.51 J678067:205-1542(+)
NODE_10042_length_1345_cov_20691.368992_g138_i17.p2	NODE_10042_length_1345_cov_20691.368992_g138_i17	5082.365988	Apamin	0.00000833	34.7	Apamin	8.83E-07	34.7	NA	NA	NA	Toxin	Apamin	40	108	NODE_10042_length_1	131.96	59	9	9	Carbamidom	5599	GENE.NODE_10042_length_1345_cov_20691.368992_g138_i17~~NODE_10042_length_1345_cov_20691.368992_g138_i17.p2 ORF type:complete len:51 (+) score=-3.30 Bee_toxin|PF17454.2|8.3e-08 NODE_10042_length_1345_co...
NODE_1035_length_5669_cov_154.173579_g758_i0.p2	NODE_1035_length_5669_cov_154.173579_g758_i0	40.89185579	NA	NA	NA	NA	NA	NA	Moesin/ezrin/radixin 1	4.49E-148	429	No venom function	NA	177	218	NODE_1035_length_56	65.85	10	2	2		27038	GENE.NODE_1035_length_5669_cov_154.173579_g758_i0~~NODE_1035_length_5669_cov_154.173579_g758_i0.p2 ORF type:complete len:228 (+) score=58.44 ERM|PF00769.19|8.2e-61 DUF2203|PF09969.9|0.0087 DUF2203|PF09969....
NODE_10529_length_1115_cov_107.359615_g7997_i0.p1	NODE_10529_length_1115_cov_107.359615_g7997_i0	57.37424136	NA	NA	NA	Histone H4	1.44E-70	199	Histone H4 (Fragment)	9.39E-67	201	No venom function	NA	73	84	NODE_10529_length_1	104.87	47	5	5	Oxidation (M	11500	GENE.NODE_10529_length_1115_cov_107.359615_g7997_i0~~NODE_10529_length_1115_cov_107.359615_g7997_i0.p1 ORF type:complete len:104 (+) score=19.69 CENP-T_C|PF15511.6|2.2e-08 TAF|PF02969.17|3.6e-05 CENP-S|P...
NODE_11081_length_1183_cov_321.804965_g7134_i1.p1	NODE_11081_length_1183_cov_321.804965_g7134_i1	0	NA	NA	NA	NA	NA	NA	Superoxide dismutase [Cu-Zn], chloroplastic	2.53E-88	287	Venom component (parasitoids)	Superoxide dismutase	110	179	NODE_11081_length_1	109.62	21	2	2	Carbamidom	18270	GENE.NODE_11081_length_1183_cov_321.804965_g7134_i1~~NODE_11081_length_1183_cov_321.804965_g7134_i1.p1 ORF type:complete len:173 (+) score=20.71 Sod_Cu|PF00080.20|1.4e-46 NODE_11081_length_1183_cov_321.8...
NODE_11225_length_1162_cov_51432.834688_g2195_i1.p4	NODE_11225_length_1162_cov_51432.834688_g2195_i1	13.00180871	Apamin	0.00000146	36.6	Apamin	1.55E-07	36.6	NA	NA	NA	Toxin	Apamin	13	115	NODE_11225_length_1	197.87	56	23	2	Carbamidom	6237	GENE.NODE_11225_length_1162_cov_51432.834688_g2195_i1~~NODE_11225_length_1162_cov_51432.834688_g2195_i1.p4 ORF type:5prime_partial len:55 (+) score=-5.37 Bee_toxin|PF17454.2|2.2e-08 NODE_11225_length_1...
NODE_11494_length_1125_cov_206.560748_g8269_i0.p1	NODE_11494_length_1125_cov_206.560748_g8269_i0	98.97275408	NA	NA	NA	NA	NA	NA	Peptidyl-prolyl cis-trans isomerase	1.44E-141	398	No venom function	NA	72	87	NODE_11494_length_1	105.47	24	5	5	Carbamidom	22861	GENE.NODE_11494_length_1125_cov_206.560748_g8269_i0~~NODE_11494_length_1125_cov_206.560748_g8269_i0.p1 ORF type:complete len:207 (+) score=26.09 Pro_isomerase|PF00160.21|5.8e-52 NODE_11494_length_1125_co...
NODE_11707_length_957_cov_56.019274_g8998_i0.p1	NODE_11707_length_957_cov_56.019274_g8998_i0	9.231161332	NA	NA	NA	NA	NA	NA	Sep15_SelM domain-containing protein	5.08E-95	276	No venom function	NA	68	75	NODE_11707_length_9	109.2	30	5	5	Carbamidom	18018	GENE.NODE_11707_length_957_cov_56.019274_g8998_i0~~NODE_11707_length_957_cov_56.019274_g8998_i0.p1 ORF type:complete len:155 (+) score=12.59 Sep15_SelM|PF08806.11|5.6e-27 NODE_11707_length_957_cov_56.01927...
NODE_12215_length_900_cov_350.141818_g8219_i1.p1	NODE_12215_length_900_cov_350.141818_g8219_i1	213.5472267	NA	NA	NA	NA	NA	NA	Superoxide dismutase [Cu-Zn], chloroplastic	2.53E-88	287	Venom component (parasitoids)	Superoxide dismutase	110	178	NODE_12215_length_9	109.62	21	2	2	Carbamidom	18270	GENE.NODE_12215_length_900_cov_350.141818_g8219_i1~~NODE_12215_length_900_cov_350.141818_g8219_i1.p1 ORF type:complete len:173 (+) score=20.71 Sod_Cu|PF00080.20|1.4e-46 NODE_12215_length_900_cov_350.14181...
NODE_12300_length_891_cov_419.590686_g9496_i0.p1	NODE_12300_length_891_cov_419.590686_g9496_i0	21.45689413	NA	NA	NA	NA	NA	NA	Superoxide dismutase [Cu-Zn]	2.37E-93	272	Venom component (parasitoids)	Superoxide dismutase	49	42	NODE_12300_length_8	150.66	61	8	8	Carbamidom	15698	GENE.NODE_12300_length_891_cov_419.590686_g9496_i0~~NODE_12300_length_891_cov_419.590686_g9496_i0.p1 ORF type:complete len:153 (+) score=17.11 Sod_Cu|PF00080.20|1.4e-51 CHRD|PF07452.12|0.056 NODE_12300_le...
NODE_12392_length_882_cov_458.973978_g9580_i0.p1	NODE_12392_length_882_cov_458.973978_g9580_i0	291.9235864	NA	NA	NA	NA	NA	NA	Transgelin	5.4E-130	367	No venom function	NA	81	89	NODE_12392_length_8	112.78	29	4	4	Carbamidom	20570	GENE.NODE_12392_length_882_cov_458.973978_g9580_i0~~NODE_12392_length_882_cov_458.973978_g9580_i0.p1 ORF type:complete len:185 (+) score=37.87 CH|PF00307.31|1.1e-23 Calponin|PF00402.18|4e-10 CAMSAP_CH|PF1...
NODE_12433_length_1006_cov_20793.932702_g1692_i3.p4	NODE_12433_length_1006_cov_20793.932702_g1692_i3	2490.904444	NA	NA	NA	NA	NA	NA	Uncharacterized/Protein CBR-GRL-22	1.20E+00	75	Unknown function	NA	2	241	NODE_12433_length_1	254.68	63	24	21	Carbamidom	5986	GENE.NODE_12433_length_1006_cov_20793.932702_g1692_i3~~NODE_12433_length_1006_cov_20793.932702_g1692_i3.p4 ORF type:complete len:54 (+) score=3.86 Bee_toxin|PF17454.2|0.14 NODE_12433_length_1006_cov_20...
NODE_1293_length_5449_cov_108.784946_g950_i0.p1	NODE_1293_length_5449_cov_108.784946_g950_i0	37.9258919	NA	NA	NA	NA	NA	NA	Carboxypeptidase D	0	2285	Venom component	CarboxypeptidaseD	133	322	NODE_1293_length_54	38.7	1	2	2		189105	GENE.NODE_1293_length_5449_cov_108.784946_g950_i0~~NODE_1293_length_5449_cov_108.784946_g950_i0.p1 ORF type:complete len:1674 (+) score=219.70 Peptidase_M14|PF00246.24|2.8e-78 Peptidase_M14|PF00246.24|8.6e...
NODE_1388_length_4997_cov_135.900244_g975_i1.p1	NODE_1388_length_4997_cov_135.900244_g975_i1	53.08652634	NA	NA	NA	Arginine kinase	0	672	Arginine kinase	0	716	Venom component	Arginine kinase	46	18	NODE_1388_length_49	159.52	30	10	10	Carbamidom	39904	GENE.NODE_1388_length_4997_cov_135.900244_g975_i1~~NODE_1388_length_4997_cov_135.900244_g975_i1.p1 ORF type:complete len:356 (+) score=62.47 ATP-gua_Ptrans|PF00217.19|6e-86 ATP-gua_PtransN|PF02807.15|2.1e-...
[illegible]	[illegible]	[illegible]	[illegible]	[illegible]	[illegible]	[illegible]	[illegible]	[illegible]	[illegible]	[illegible]	[illegible]	[illegible]	[illegible]	[illegible]	[illegible]	[illegible]	[illegible]	[illegible]	[illegible]	[illegible]	[illegible]	[illegible]	[illegible]
